# Transcriptome, metabolome and suppressor analysis reveal an essential role for the ubiquitin-proteasome system in seedling chloroplast development

**DOI:** 10.1186/s12870-022-03536-6

**Published:** 2022-04-08

**Authors:** Prabhavathi Talloji, Lilian Nehlin, Bruno Hüttel, Nikola Winter, Martin Černý, Hana Dufková, Bulut Hamali, Katarzyna Hanczaryk, Jan Novák, Monika Hermanns, Nicole Drexler, Karolin Eifler, Nikolaus Schlaich, Břetislav Brzobohatý, Andreas Bachmair

**Affiliations:** 1 Department of Biochemistry and Cell Biology, Max Perutz Labs/Center for Molecular Biology, University of Vienna, A-1030 Vienna, Austria; 2 Max Planck Genome Centre Cologne, Max Planck Institute for Plant Breeding Research, 50829 Cologne, Germany; 3 Department of Molecular Biology and Radiobiology, Faculty of AgriSciences, Mendel University in Brno, CZ-613 00 Brno, Czech Republic; 4 Present address: Department of Integrative Biology, Oregon State University, 3029 Cordley Hall, Corvallis, OR 97331 USA; 5 Institute of Plant Physiology (Bio III), RWTH-Aachen, 52056 Aachen, Germany; 6 Vienna Biocenter Core Facilities, Electron Microscopy, A-1030 Vienna, Austria; 7 CEITEC – Central European Institute of Technology, Mendel University in Brno, CZ-61300 Brno, Czech Republic

**Keywords:** Ubiquitin K48 chains, Chloroplast development, Photomorphogenesis, Phototropin, Light signal transduction, Light stress, Chlorophagy

## Abstract

**Background:**

Many regulatory circuits in plants contain steps of targeted proteolysis, with the ubiquitin proteasome system (UPS) as the mediator of these proteolytic events. In order to decrease ubiquitin-dependent proteolysis, we inducibly expressed a ubiquitin variant with Arg at position 48 instead of Lys (ubK48R). This variant acts as an inhibitor of proteolysis via the UPS, and allowed us to uncover processes that are particularly sensitive to UPS perturbation.

**Results:**

Expression of ubK48R during germination leads to seedling death. We analyzed the seedling transcriptome, proteome and metabolome 24 h post ubK48R induction and confirmed defects in chloroplast development. We found that mutations in single genes can suppress seedling lethality, indicating that a single process in seedlings is critically sensitive to decreased performance of the UPS. Suppressor mutations in phototropin 2 (*PHOT2*) suggest that a contribution of *PHOT2* to chloroplast protection is compromised by proteolysis inhibition.

**Conclusions:**

Overall, the results reveal protein turnover as an integral part of a signal transduction chain that protects chloroplasts during development.

**Supplementary Information:**

The online version contains supplementary material available at 10.1186/s12870-022-03536-6.

## Background

Responses of plants to environmental or developmental cues frequently require the covalent attachment of small protein modifier ubiquitin to substrate proteins [1, 2]. Targeted destruction of key regulators upon ubiquitin conjugation, as a response to stimuli, is amply documented [1, 3–5]. In this reaction, the carboxyl-terminal Gly residue of ubiquitin is linked to the active site Cys of ubiquitin activating enzyme E1, to form a thioester bond. Transfer of ubiquitin to the Cys residue of a ubiquitin conjugating enzyme E2 is usually followed by ubiquitin conjugation to Lys side chain amino groups of substrate proteins. Formation of these isopeptide bonds requires a ubiquitin ligase, E3 [6, 7]. A prominent class of multisubunit E3s are the cullin-RING ligases. Ligases based on the cullin 3 scaffold contain a BTB/POZ domain substrate recognition subunit [8, 9]. Interestingly, previous work has defined two distinct functions for proteins with BTB domain. One is to serve as substrate adaptors for a cullin 3 ligase, which implies participation in turnover [10, 11]. The second function is interaction with other BTB proteins to (hetero)dimerize [12, 13]. The latter interaction could sequester or even destabilize [14] subunits of active ligases, i. e. prevent turnover. Examples for BTB/POZ domain proteins are NPH3 (NON-PHOTOTROPIC HYPOCOTYL 3), a component of phototropin signalling, and its relatives [15].

NPH3 and other family members [15, 16] were shown to bind phototropins 1 and/or 2, but rapid turnover was only found for phot1, not for its close relative phot2 (we use italics for the phototropin WT gene, large print for the apoprotein, and small print for the protein with chromophore). phot1 and phot2 are structurally related, but functionally distinct, with phot2 being responsible for a number of responses to high light intensity [17, 18]. In particular, phot2 mediates chloroplast re-positioning to protect them from excess light. Other roles in prevention of light damage, or a contribution to de-etiolation or chloroplast maintenance, has not been reported so far in higher plants. This contrasts with the single phot protein of Chlamydomonas, which has a critical role in chloroplast maintenance, by modulating gene expression to protect chloroplasts from light damage [19]. Interestingly, higher plant phot2 localizes both to the plasma membrane and to the chloroplast. However, no function for the chloroplast-localized fraction has been identified so far [20], indicating that additional phot2 functions remain to be discovered.

Ubiquitin is frequently conjugated to ubiquitin, resulting in a chain of several ubiquitin moieties linked to the substrate. All seven Lys residues of ubiquitin are used as ubiquitin attachment sites in chain formation. However, the importance of these linkage types varies. In budding yeast, replacement of ubiquitin Lys 48 by Arg to prevent formation of ubiquitin chains linked via Lys 48 is lethal, whereas all other single replacements are compatible with viability [21, 22]. Indeed, Lys 48 is the most abundant ubiquitin attachment site in all eukaryotes including plants [23]. Substrates decorated with ubK48 chains are channelled into proteasomal degradation. In order to study ubiquitin- and proteasome-dependent processes, we have introduced into Arabidopsis a transgene for inducible expression of a ubiquitin variant with Arg instead of Lys at position 48 (ubK48R). In vitro, this variant allows monoubiquitylation and formation of ubiquitin chains, except for those linked via Lys 48. Induction of the ubiquitin variant ubK48R in Arabidopsis decreases turnover of key cellular regulators such as cyclin and phytochrome A [24, 25]. Expression of ubK48R during germination leads to death at seedling stage.

Chemical inhibition of the UPS via proteasome inhibitors also leads to cessation of growth usually followed by cell death in animals [26], yeast [27] and in plants [28]. Proteasome inhibition in plants has been found to down-regulate translation and results in starch accumulation [29, 30]. Similarly, the UPS is important in the response to light stress, as demonstrated via chemical inhibition, and via mutagenesis of proteasomes [31, 32]. Also relevant in this context, protein import into chloroplasts and chloroplast quality control are guarded by ubiquitin ligases [33, 34], with impact on developmental transitions.

In this work, we analyzed changes that occur in response to ubK48R expression in Arabidopsis. Compared to chemical inhibition of the proteasome, we detected both similarities and differences. Apart from differences in the time scale of experimentation (hours of proteasome inhibitor exposure versus 1 day ubK48R induction), one feature of ubK48R expression may contribute to the specifics of the approach presented in this work: The ubK48R variant leads to shortening of ubiquitin K48 chains, a manipulation that affects proteolysis substrates with long and complex ubiquitin chains more than those decorated with short chains. In mammals, linkage of many ubiquitin moieties to a substrate, usually in the form of several or even branched chains, correlates with very fast turnover rates [35–37]. For this reason, ubK48R expression at moderate levels might be selectively blocking fast turnover of the most short-lived proteins.

We found that developing chloroplasts were degraded when the ubK48R transgene was induced in germinating seedlings. Chloroplast development is guided by light reception [19, 38–40]. A screen for suppressors of the lethal effects of ubK48R expression indicated that mutations in an aspartate protease, in the NPH3 family BTB/POZ domain protein NRL16, and in *PHOT2* suppressed lethality and allowed chloroplast development. The nph3 homolog nrl16 and PHOT2 interact in yeast two hybrid assays, suggesting that they may operate in the same signal transduction pathway. These components may perform a previously undescribed function in higher plants, protecting chloroplasts during development.

## Results

**Fig. 1:**
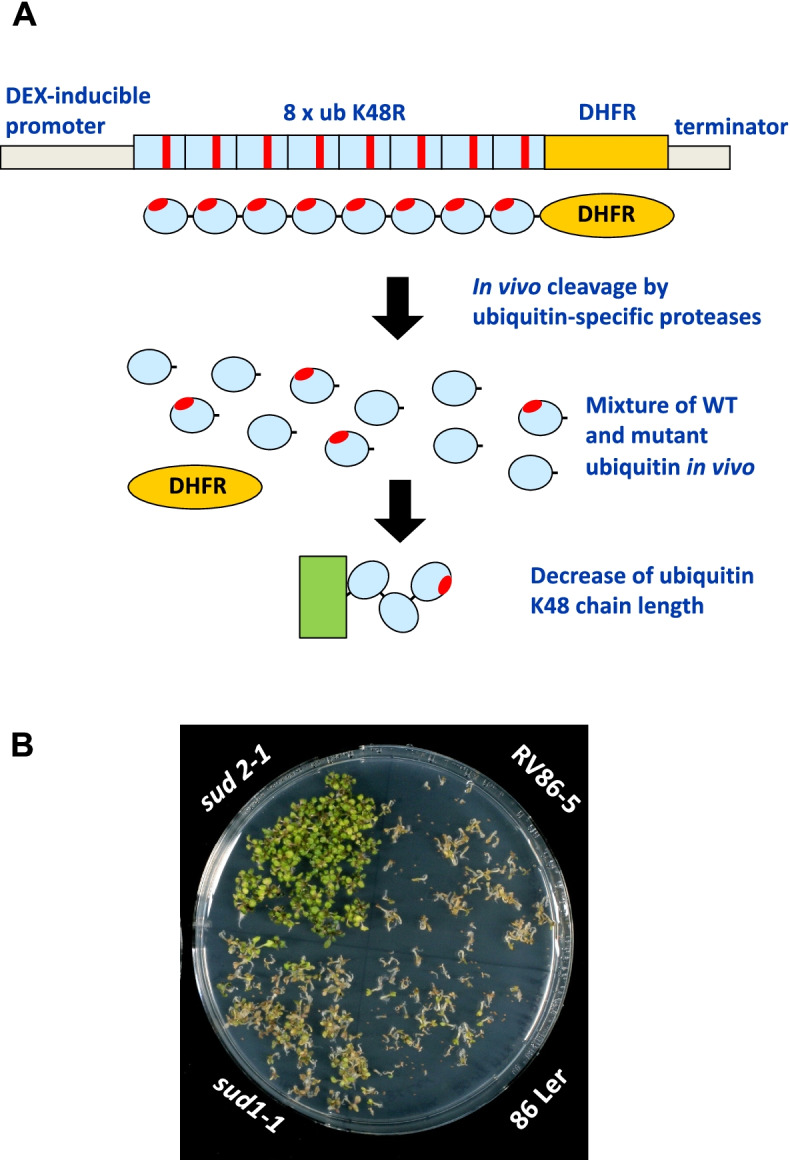
**A** A ubK48R transgene can be induced by Dexamethasone (DEX). The transgene contains one open reading frame encompassing eight ubiquitin units, each with a Lys to Arg change at position 48, followed by a methotrexate resistance gene (murine dihydrofolate reductase, DHFR). Induction of the transgene decreases the average length of ubiquitin K48 chains, thereby decreasing ubiquitin-dependent protein turnover, which leads to growth arrest and cell death. **B** Plate assay for death of line RV86–5 and a line with the same transgene introgressed into L*er* (86 Ler). Whereas these two lines die in presence of inducer DEX, EMS-induced mutations in single mendelian loci could prevent seedling death, as shown for mutants *sud1–1* and *sud2–1*

Expression of ubiquitin variant ubK48R inhibits the ubiquitin proteasome system (UPS) in seedlings [24] and results in seedling lethality. Figure 1 shows the expression scheme and a standard assay with the ubK48R transgene induced in seedlings by dexamethasone (DEX) presence in plates, either in Col-0 plants (RV86–5), or after introgression of the transgene into the L*er* background (86 Ler). Figure 1 also shows growth of two suppressor lines generated by EMS mutagenesis (*sud1–1*, *sud2*–*1*; for details, see below). Inhibition of the UPS has lethal consequences in all eukaryotes tested so far [26–28, 41] (see also Note S1 and Figs. S1 and S2).

### Changes of transcription after ubK48R expression impact on chloroplast development

To gain more insight into changes occurring upon ubK48R expression, transcriptional profiling was carried out by RNA analysis from (ubK48R transgenic) RV86–5 seedlings exposed to DEX for 24 h. RNA from non-exposed seedlings served as reference RNA source. The ATH1 chip hybridization procedure gave results as summarized in Table 1. The extended data set of up- and down-regulated genes can be found in Tables S1 and S2. Induction of the inhibitory ubiquitin ubK48R variant leads to up-regulation of 780 genes more than 2.4-fold. In parallel, 855 genes are down-regulated more than 2.4-fold. Thus, the number of significantly up-regulated and the number of significantly down-regulated genes are approximately equal (ratio 0.9). When searching for GO terms `biological process´ that are specifically enriched, it turns out that up-regulated genes have a broader distribution (and thus less specific enrichment) than down-regulated genes. For instance, the most highly enriched term for up-regulated genes is `response to wounding´ with 3.65-fold enrichment, whereas other terms are enriched only up to 2.52-fold. This contrasts with the set of down-regulated genes, where terms such as `protein targeting to chloroplast´, `chloroplast organization´, `photosynthesis´, or `response to light stimulus´ are enriched between 8.29- and 4.61-fold. Somewhat less highly enriched in the set of down-regulated genes are terms of ribosome biogenesis (5.21- to 3.83-fold enrichment). The statistics of GO `biological process´ terms are summarized in Table S3. Characteristic features of the gene expression data with particular relevance to this work are as follows (for additional descriptions, see Notes S2).

**Table 1:** Some characteristic changes in gene regulation after ubK48R induction

Category	up-regulated	down-regulated	ratio up vs. dn
Total^a^	780	855	0.9
Ubiquitin conjugation	20	22	0.9
Transcription, translation	4	70	0.1
Chloroplast components	37	234	0.2
Mitochondrial components	60	21	2.9
Red-ox homeostasis	16	40	0.4
Hormonal growth promotion	21	34	0.6
Defense related	44	47	0.9
Cell wall remodeling	45	31	1.5
Phosphorylation	36	23	1.6

^a^The list does not include all transcripts, so that the sum of the mentioned transcripts does not add up to the total number

#### Translational apparatus (ribosome biogenesis, mRNA processing etc.)

There was a clear down-regulation of the translation machinery. This included ribosome biogenesis and mRNA maturation. Fifty-seven genes of this category were down-regulated, whereas only 9 genes of this category were up-regulated. In addition, chaperone gene expression was decreased. Thirteen genes of cytosolic or ER-resident chaperones were down-regulated, but only four were up-regulated. These changes are consistent with the observed cessation of growth. Interestingly, down-regulation of translation is also one of the most prominent early consequences of proteasome inhibition, starting probably before changes in transcription can take effect [29, 42]. Down-regulation of translation may therefore rely on both post-transcriptional, and on transcriptional adjustments.

#### Organelle targeted or encoded genes

Two hundred thirty-four genes encoding chloroplast components were down-regulated more than 2-fold (Table S2). These genes include components of plastid transcription and translation, of amino acid biosynthesis, of the photosynthetic apparatus, and chaperones. Only 37 genes encoding chloroplast components were up-regulated. In contrast, 21 genes with products residing in mitochondria were down-regulated (6- to 2.4-fold)**,** whereas 60 were up-regulated (8- to 2.4-fold)**.**

Therefore, ubK48R-induced seedlings do not develop their chloroplasts like WT. The specific decrease in transcript abundance for chloroplast factors, on top of the general down-regulation of translation, indicates that fewer proteins are imported or available for import. In contrast, mitochondrial components are up-regulated, suggesting ongoing support for, but also adjustments in mitochondrial activity. Seedlings used in the experiments were grown on sucrose-supplemented media and should therefore not be limited in carbohydrate availability. We speculate that the opposing regulation of mitochondrial components may be installed to maintain basic metabolism under low light conditions or upon otherwise compromised chloroplast function when plants grow on soil. Next, we searched for indications that plants may be compromised in light perception that normally guides chloroplast development. Interestingly, the transcription profile offered hints that there were changes in blue light signalling.

#### Light signalling

Regarding photoreceptor-dependent signalling, only blue light reception was significantly altered at the transcriptional level: transcription from *PHOT1* was down 2.7-fold, from *PHOT2* 3.5-fold; NPH3 (NON-PHOTOTROPIC HYPOCOTYL3) and two NPH3 family proteins (NRL12, At3g15570; NRL13, At3g19850) were down-regulated, as well as another protein known to form a complex with phot1 and phot2 (phytochrome kinase substrate 2, At1g14280). The light regulatory transcription factor HY5 homolog (HYH) was down-regulated 3-fold. Likewise, transcription factor RHL41, with assigned role in high light adaptation, was also down-regulated 3-fold. No component of blue light reception was up-regulated. In contrast, no red light-response component was down-regulated (HYH is co-regulated by phytochromes, though), only one red light signal transduction component, FRE1, was mildly up-regulated (2.6-fold). We therefore hypothesized that light regulation, and specifically blue light signalling via phototropin, was affected by ubK48R induction.

### Proteome changes after ubK48R induction

Two-week old seedlings grown on sucrose medium were also used for proteome analysis. Growth and induction were similar to the transcriptome analysis, encompassing a 24 h-induction with DEX. A total of 3220 proteins, representing over 1800 protein groups, were identified by stringent criteria (see Table S4). Among all 95 proteins with significant abundance changes, 31 were from chloroplasts and reduced in abundance, only eight chloroplast proteins increased in abundance. The data confirm and extend the transcriptome data by indicating down-regulation of a number of chloroplast proteins (for additional details, see Notes S3).

### Changes in metabolites after ubK48R induction

**Fig. 2:**
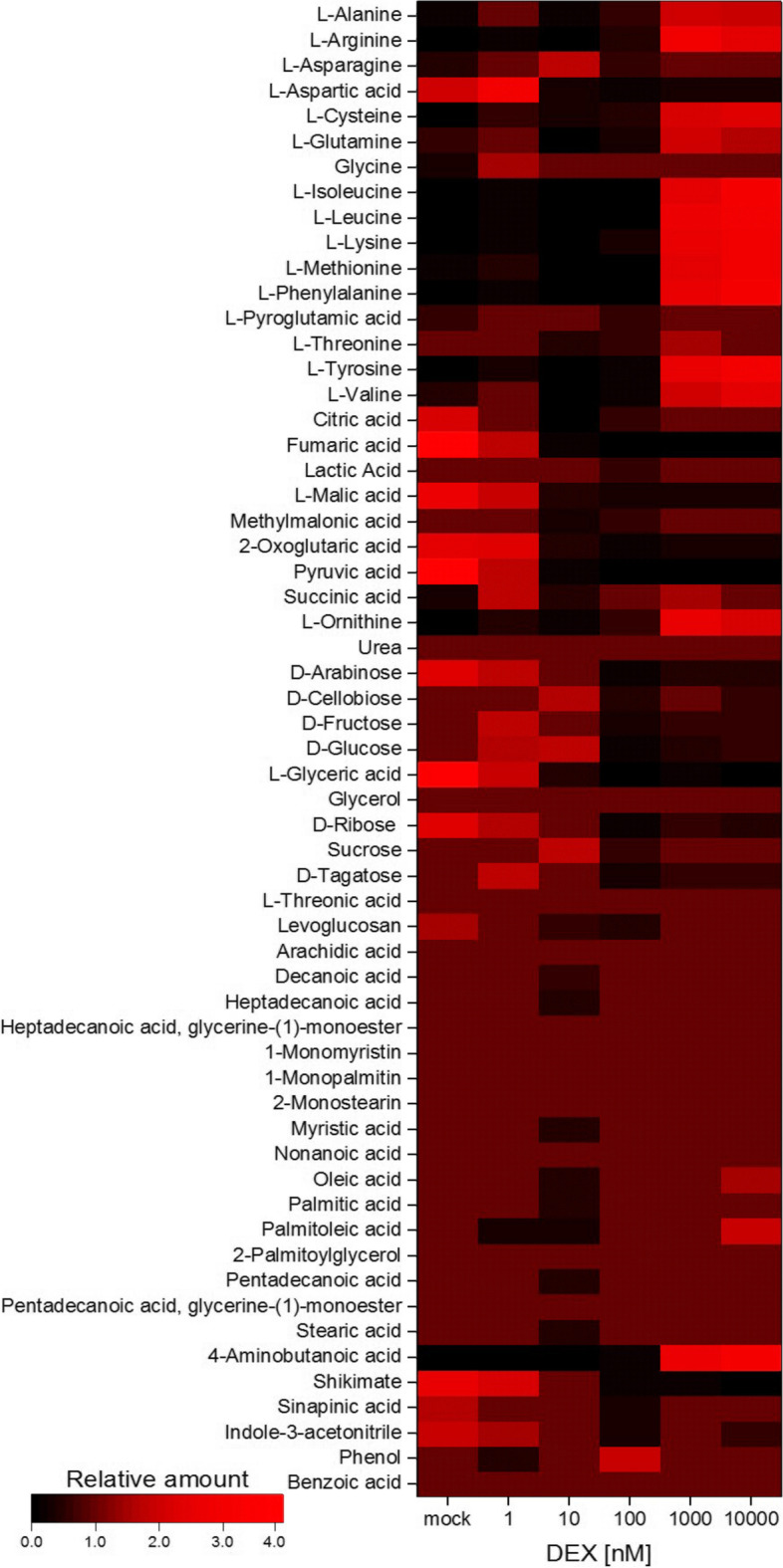
Changes in key metabolites upon induction of the ubK48R transgene in seedlings

Most important results of a metabolome analysis are shown in Fig. 2 and in Table S5. Of note, ubK48R induction causes a decrease of tricarboxylic acid cycle metabolites, in particular citric acid, fumaric acid, malic acid and 2-oxoglutarate. This change again points to alterations in mitochondria, as concluded from the transcriptome data. Furthermore, an observed increase in intracellular glucose, fructose and sucrose supports the interpretation that lack of energy per se is not a cause for down-regulation of protein synthesis. Thirdly, higher DEX concentrations (see Methods S4) led to accumulation of a broad spectrum of amino acids. Therefore, amino acid shortage due to lack of protein recycling is not causative for the phenotype of ubK48R induction in seedlings (cf. Figs. S1 and S2, Notes S1).

To summarize, transcriptome and proteome analyses indicated that major growth-related, energy requiring processes such as translation and chloroplast development were down-regulated upon ubK48R induction. However, given the constraints of decreased translation, it is not obvious why chloroplast-related mRNAs are specifically down-regulated, even though chloroplast functioning is a precondition for survival at the seedling stage. It is therefore possible that specific interference with chloroplast development contributed to down-regulation of so many chloroplast-related transcripts. We employed suppressor analysis in an attempt to address this issue.

### Mutations in single genes can suppress ubK48R-induced phenotypes

We have previously reported that EMS mutagenesis of line RV86–5 resulted in the isolation of mutations with mendelian segregation pattern that are able to (partially) suppress growth arrest and lethality after ubK48R induction [24]. Such suppressor genes were called **S**UPPRESSORS OF **U**BK48R-INDUCED CELL **D**EATH (SUD). Figure 1 shows growth of two suppressor lines obtained via EMS mutagenesis, *sud1–1* and *sud2–1*, in comparison with the non-mutagenized RV86–5 line. Table 2 summarizes suppressor mutations discussed in this work.

**Table 2:** Suppressor mutations discussed in this work

Identifier	designation	allele characteristic	functional annotation	reference
Unknown	*sud1–1*	dominant, EMS-induced	unknown	ref. [24]
Unknown	*sud2–1*	recessive, EMS-induced	unknown	this work
At1g62290	*sud7–1*	T-DNA insertion	aspartate protease	this work
		FLAG_554E08		
At3g44820	*nrl16–2*	T-DNA insertion	BTB/POZ protein	this work
		SALK_143045	potential ubiquitin	
			ligase subunit	
At5g58140	*phot2–81*	1 bp deletion (frame shift)	blue light receptor	this work
At5g58140	*phot2–82*	2 bp deletion (frame shift)	blue light receptor	this work
At5g58140	*phot2–83*	9 bp deletion (in frame deletion)	blue light receptor	this work

**Fig. 3:**
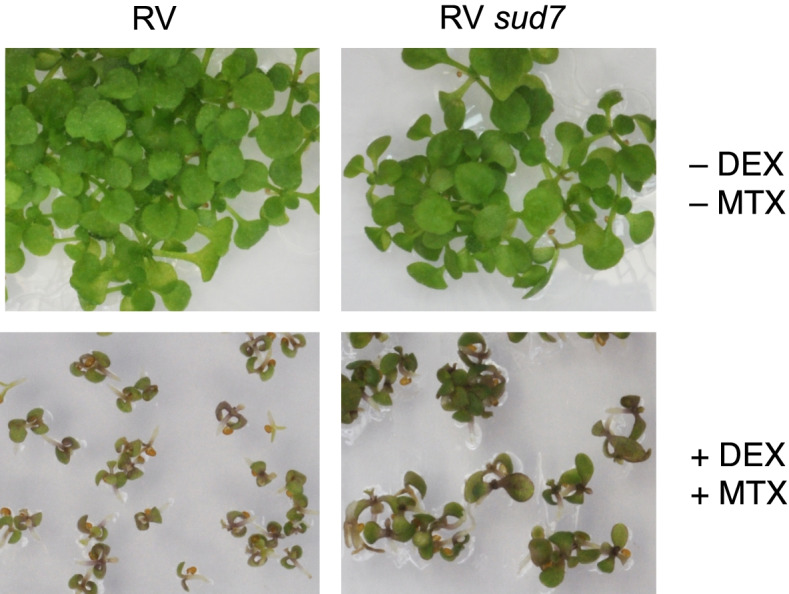
Growth arrest and death of RV86–5 seedlings (RV) is suppressed by a T-DNA insertion in gene SUD7 in presence of inducer DEX and selection agent methotrexate (MTX)

To find out whether any of those genes that are highly induced after ubK48R induction were causally linked to failed chloroplast development, growth arrest, or cell death, we employed suppressor mutant *sud2–1*, which segregates as a single recessive trait in crosses. Transcriptome comparison of the RV86–5 line with the RV86–5 *sud2–1* line after parallel induction by DEX pointed to 38 genes that were significantly higher expressed in RV86–5 than in RV86–5 *sud2–1* (for the data set, see Table S6). T-DNA insertions for nine of these genes were introgressed into the RV86–5 background for testing. Mutation in one of these genes, a putative saposin-like aspartyl protease (At1g62290, also listed as PASPA2), at least partially suppressed growth arrest and cell death (Fig. 3). The gene was named SUD7. The *sud7* allele tested in this work was generated in the Ws genetic background, crossing to line RV86–5 therefore resulted in a mixed genetic background. However, the RV86–5 transgene is effective in different ecotypes (Fig. 1), so that we do not assume that the mixed genetic background significantly influenced these results. SUD7 is highly expressed during embryo development and germination (transcriptome data accessed via GENEVESTIGATOR). Interestingly, SUD7 showed increased expression in a number of experiments with imbalance between light exposure and chloroplast development. For instance, exposure of a phytochrome (quintuple) mutant, or of phytochrome interacting factor (quadruple and double) mutants to light induces SUD7 compared to WT [43–45] (ATH1 chip expression data AT-00601, AT-00526, AT-00518, AT-00246, of GENEVESTIGATOR). Similarly, treatment to block chloroplast ribosomes in seedlings with lincomycin [39], sulfamethoxazole [46, 47], or an albino-phenotype causing substance (expression data AT-00501, AT-00632, AT-00639 of GENEVESTIGATOR) in presence of light induces SUD7. We therefore speculate that this gene might have a role in response to (light-)damage of chloroplasts.

**Fig. 4:**
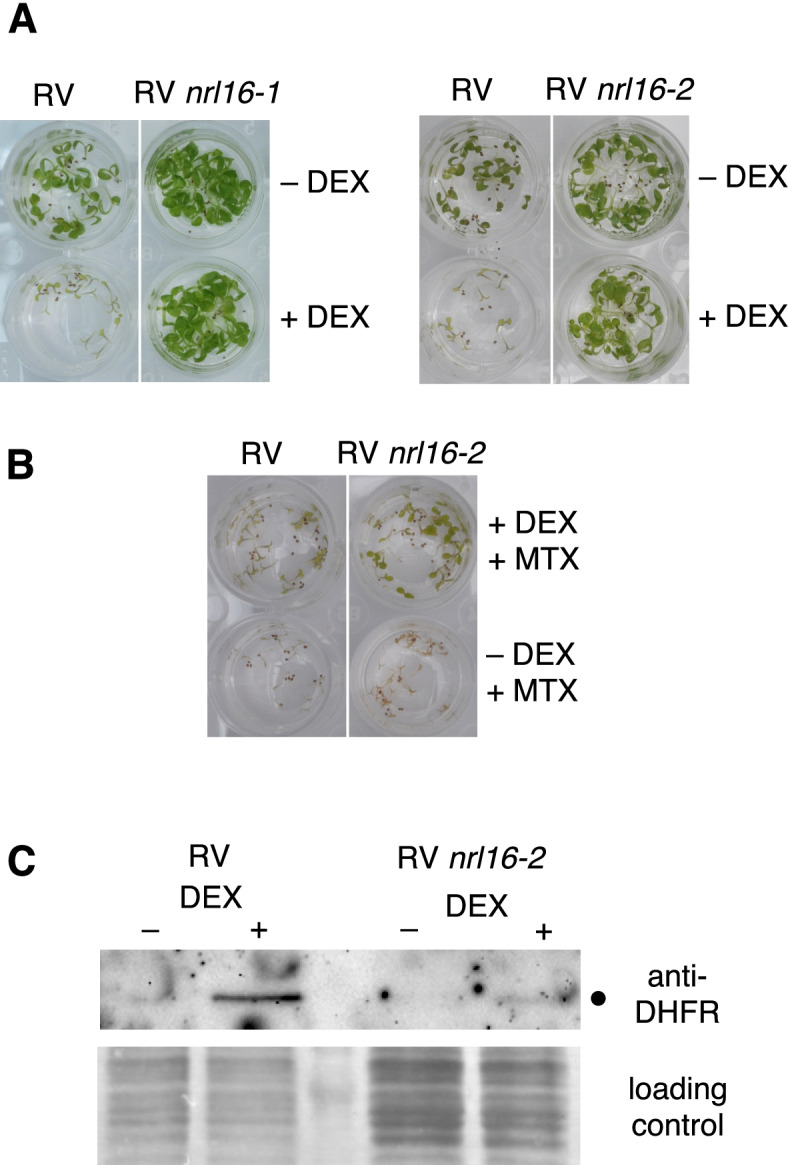
Mutations in NRL16 suppress the cell death caused by ubK48R induction. **A** Growth of the RV86–5 line is significantly inhibited by Dexamethasone (DEX) application to induce the ubK48R transgene. In contrast, lines with T-DNA insertion in NRL16 can grow almost un-impeded. **B** The same is true if methotrexate (MTX) is added to the medium. MTX alone kills both RV and the RV *nrl16–2* line immediately after germination (bottom wells), whereas induction of the ubK48R transgene with the co-expressed MTX-resistant DHFR mitigates MTX toxicity and allows some growth. Again, the line with T-DNA insert in NRL16 shows better growth. **C** *nrl16–2* mutant plants show lower expression of the ubK48R transgene (demonstrated by the level of DHFR co-translated with the ubK48R units, dot in upper panel). The transgene is, however, expressed at sufficient levels to mitigate MTX toxicity, as shown in panel B. All liquid media contain silencing inhibitor Zebularine

The gene region of the *sud2–1* mutation contains one gene with potential connection to both the UPS, and to light signalling: NRL16 (**N**PH3, **R**PT2 **L**ike gene 16, At3g44820, a potential subunit of a cullin 3 ligase). NRL16 is a homolog of NPH3 (**N**ON-**P**HOTOTROPIC **H**YPOCOTYL 3), a gene of phototropin signal transduction [16, 48]. Two independent T-DNA insertions in this gene were crossed into the RV86–5 background, one of which (*nrl16–2*) apparently disrupts an exon. We noticed, however, that the T-DNAs of both tested alleles silenced the RV86–5 transgene, so that the standard suppressor assay could not be applied. Therefore, a liquid assay including DNA methylation inhibitor Zebularine [49] was used, to counteract RV86–5 transgene silencing by NRL16-localized T-DNAs. Experiments to assess the expression level of the induced ubK48R gene indicated that the expression was not as high as in the control line without NRL16-inserted T-DNA (as evidenced by reduced abundance of the co-translated DHFR, Fig. 4C). Lower levels of ubK48R could mitigate growth inhibition, and suppressor activity of *nrl16* loss-of-function alleles may therefore not be as strong as suggested by Fig. 4.

**Fig. 5:**
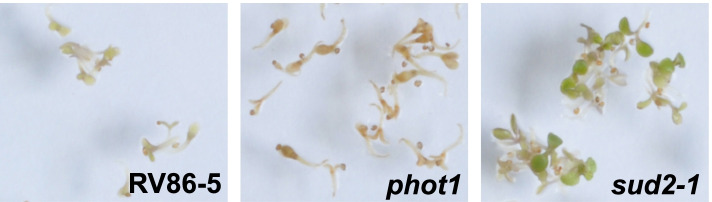
Growth of RV86–5 vs. RV86–5 *phot1* vs RV86–5 *sud2–1* on plates containing DEX and MTX

**Fig. 6:**
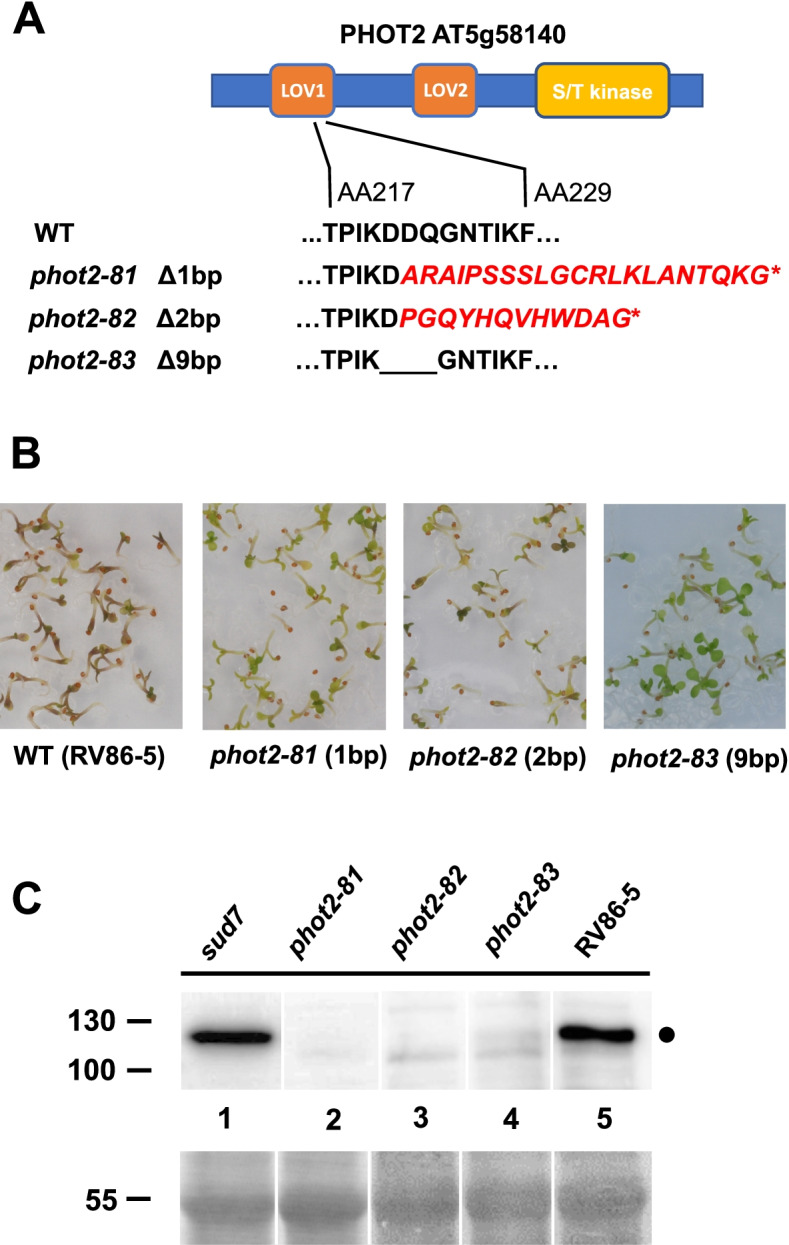
Mutations in phototropin 2 can act as suppressors of ubK48R-induced growth arrest, allowing to proceed further with photomorphogenesis and growth. **A** Mutations generated via CRISPR-Cas9 and ensuing amino acid changes. *phot2–81* is a 1-bp deletion allele, *phot2–82* a 2-bp deletion, and *phot2–83* is a 9-bp (in frame) deletion. **B** Images of growth of RV86–5 vs. RV86–5 *phot2–81,* RV86–5 *phot2–82 and* RV86–5 *phot2–83* seedlings on plates containing DEX. **C** Western blot detection of phototropin 2 in mutant and WT seedlings. RV86–5, RV86–5 *phot2–81,* RV86–5 *phot2–82*, RV86–5 *phot2–83* and in *RV86–5 sud7* seedlings were harvested after exposure to DEX. Frameshift mutations *phot2–81* and *phot-82* contain significantly reduced levels of phototropin 2, the *phot2–83* mutant contains reduced but detectable amounts of this three amino acid deletion allele. Dot, position of phototropin 2. Bottom panels: rubisco large subunit loading control as visualized by Ponceau S staining of the filter

In order to reveal connections between transcriptional down-regulation of phototropins via ubK48R and failed chloroplast development, we investigated whether mutations in phototropins impact on de-etiolation and survival after ubK48R induction. A T-DNA insertion mutant in *PHOT1* (SAIL_147_B12) was therefore combined with the RV86–5 transgene. This mutation did not prevent seedling death. If anything, the phenotype was more pronounced (Fig. 5). When a T-DNA insertion mutant in *PHOT2* (SALK_034013) was crossed with the RV86–5 transgene, homozygous progeny could not be obtained, presumably because the transgene locates close to *PHOT2*, preventing recombination. For this reason, and to circumvent potential ubK48R transgene silencing by a second T-DNA insertion, mutations in *PHOT2* were generated with the CRISPR/Cas9 system de novo, in the RV86–5 background. gRNA ACTCCTATCAAGGACGACCA, binding to the second exon that is part of the LOV1 domain, gave the best results. After outcrossing to remove the Cas9 construct, a one base pair deletion, a two base pair deletion, and a nine base pair deletion allele were used in further experiments (Table 2, Fig. 6A). Both frame shift mutations allowed greening of seedlings (Fig. 6B). There was a notable reduction of anthocyanin, and expansion of cotyledons was more pronounced. Most plants developed first leaves before an apparent growth arrest. This suggests that the suppressive power of these mutations was not as pronounced as that of *sud2* or *sud7*. However, a nine base (three amino acid) deletion mutation in *PHOT2* was a significantly stronger suppressor. The affected LOV1 domain is involved in kinase activation and dimerization [50, 51]. The phot2–81 and phot2–82 proteins are barely detectable and presumably represent complete loss of function, whereas phot2–83 can be detected at reduced levels compared to the WT (Fig. 6C; see also Fig. S4; phot2–83 protein abundance was 30% of the WT phot2 level as determined by quantitation of Western blots, std. dev. 15, average of five biological replicates). One possible explanation for better suppression of lethality by *phot2–83* is that the three amino acid deletion makes critical signal transduction complexes containing phot2 less stable, so that transition towards activation can also occur when UPS activity is reduced.

### nrl16, PHOT2 and nph3 interact in the yeast two hybrid assay

**Fig. 7:**
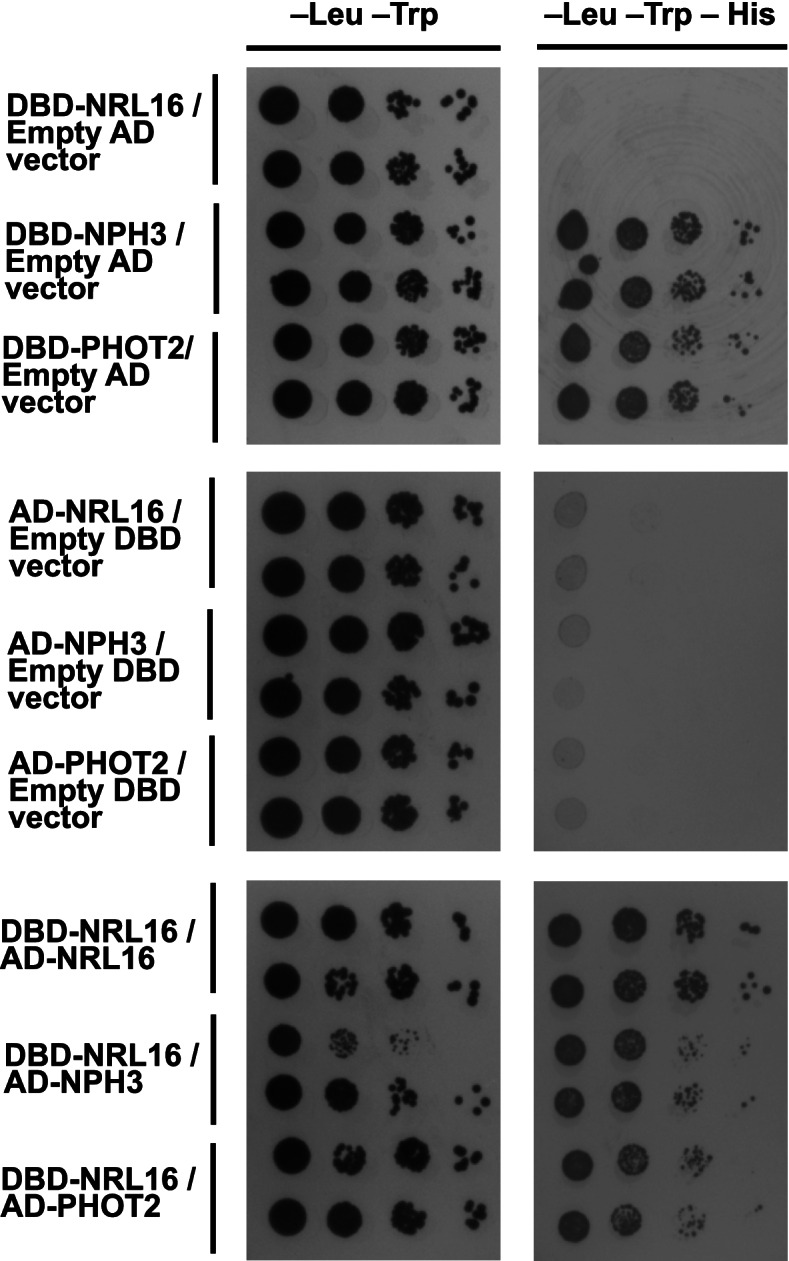
Yeast two hybrid assay demonstrates homo-dimerization of nrl16 and hetero-dimerization of nrl16 with the two blue light signal transduction components PHOT2 and nph3. Left panels: growth on plates without Leu and Trp; right panel: growth on plates without Leu, Trp and His. Two independent transformants were plated for each vector combination

We then asked whether there is a direct interaction between nrl16 and PHOT2. nph3 was included in the yeast two hybrid assay. The results of Fig. 7 show that both nph3 and PHOT2 can bind to nrl16, and nrl16 can form homodimers. Tests with nph3 and PHOT2 could be carried out only as “prey”, in combination with nrl16-DBD as a bait, due to auto-activation of PHOT2 and nph3. Nonetheless, the data are consistent with nrl16 participation in phototropin 2 signal transduction in seedlings.

### Disintegration of chloroplasts in ubK48R-induced plants

**Fig. 8:**
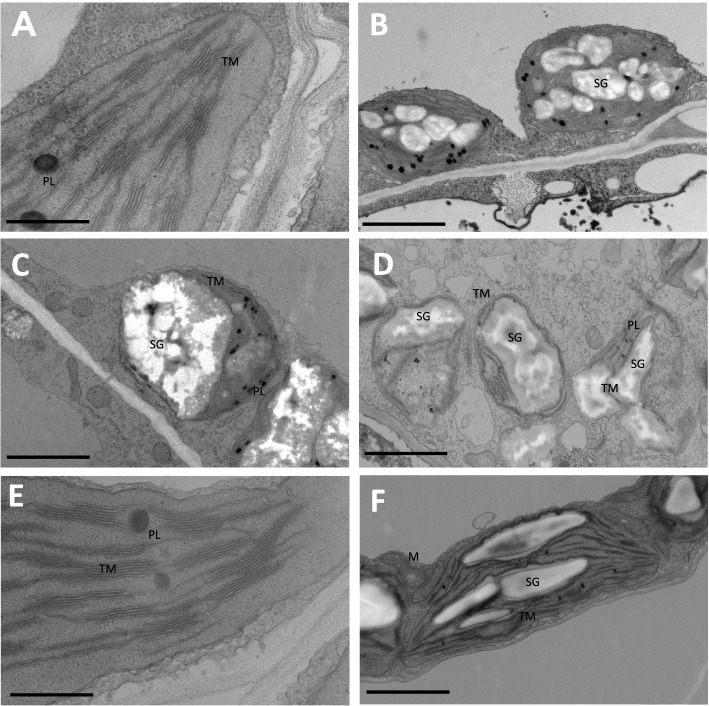
Ultrastructure of mesophyll chloroplasts from seedling cotyledons. Chloroplast development is similar in RV86–5 (**A**) and in the suppressor line RV86–5 *phot2–83* (**E**) without induced ubK48R transgene. Induction of ubK48R results in disintegration of chloroplasts in RV86–5 (**B** to **D**), whereas chloroplasts remain intact in RV86–5 *phot2–83* (**F**). Scale bars are 0.5 μm in panels **A** and **E**, and 2 μm in the other panels. SG, starch granules; PL, plastoglobuli; TM, thylakoid membranes; M, mitochondria

While it is clear from macroscopic observation that ubK48R-induced plants fail to de-etiolate, we wanted to find out whether the ultrastructure of chloroplasts allows further conclusions regarding the mechanism. For instance, etioplasts of germinating seedlings might simply not undergo any development. Alternatively, some steps of chloroplast development might occur, with subsequent deviations. We studied, via electron microscopy, sections from ubK48R-induced WT plants, and from plants with the additional *phot2–83* (3 amino acid in-frame deletion) suppressor mutation. The sections show that, upon ubK48R induction, chloroplasts develop thylakoids, but are then destroyed (Fig. 8). In contrast, the suppressor gene prevented much of the damage. An increased accumulation of starch in the chloroplasts did also occur, but may be unrelated to the death process, as it was also observed in the suppressor mutant (Fig. 8 and Fig. S5). An interpretation of these data is that the developing chloroplasts of induced seedlings are subject to processes that normally occur to remove light-damaged chloroplasts.

## Discussion

If ubiquitin proteasome system (UPS) performance decreases, several essential processes are suffering in plants [29, 32–34]. In this work, we underscore that chloroplast development of seedlings (de-etiolation) is critically affected, and point to genes that may mediate this effect. Induction of a ubiquitin variant, ubK48R, that specifically interferes with protein turnover, but allows many other ubiquitylation reactions to proceed uninhibited, can be expected to exert a broad influence on protein turnover, although proteins that require a long ubiquitin chain for efficient turnover by the proteasome might be more severely affected (Fig. 1). The expression level provided by a ubK48R octa-ubiquitin gene under control of the dexamethasone-inducible GVG transcriptional activator [52] is sufficient to stop growth and cause seedling death (octa-ubiquitin is cleaved into eight single ubiquitin moieties after translation due to the presence of ubiquitin-specific proteases in the cytoplasm; Fig. 1). Changes observed are the consequence of ubK48R expression, as opposed to side effects of transcriptional activator GVG, although previous work had indicated that elevated levels of GVG can inhibit growth [53]. Comparison to a GUS transgene (Fig. S6) and to an activated protein kinase [54], all expressed from the promoter used in this work, led to the conclusion that the major effects investigated in this work are due to ubK48R (Notes S4). We also made sure that lethality of ubK48R expression is not a direct result of amino acid shortage, which sets in after UPS inhibition in animals and in yeast [41] (see Notes S1, Figs. S1, S2). In order to understand which essential processes are compromised in the seedlings, the transcriptome and proteome were analyzed.

### Proteolysis inhibition impacts on chloroplast development in seedlings

Altered transcription patterns pointed to deep changes in cellular physiology (Tables S1, S2). The most pronounced effect of ubK48R expression is a coordinated, significant down-regulation of 234 genes for chloroplast-targeted or -encoded proteins. Down-regulation of chloroplasts and other changes reflected in the transcription pattern were supported by proteome analysis (Table S4).

Chloroplast down-regulation can be expected to decrease energy supply. A coordinated up-regulation of ca. sixty genes encoding mitochondria-targeted proteins presumably allows mitochondrial respiration to satisfy energy requirements. In parallel, global protein synthesis is coordinately down-regulated, which has been described previously as an early response to proteasome inhibition [29, 42]. In the somewhat longer treatment applied here, we find transcriptional down-regulation of genes necessary for ribosome biogenesis, other components of the translation apparatus, and genes of protein folding catalysis. However, seedlings used in these experiments were grown on sucrose-supplemented agar media, and intracellular levels of sucrose, glucose and fructose increase upon ubK48R induction (Fig. 2, Table S5). Induced plants are therefore not limited in carbohydrate availability, both for growth and for energy generation. The regulatory circuits triggered by the experiment may thus exist to cope with decreasing chloroplast performance under natural conditions, where external sugar is not available.

### Indications that light perception or light response might be mis-regulated

An interpretation of these changes is that ubK48R expressing plants have problems tuning chloroplast development to the available light. It is known that several light sensors (in particular, phyA, cry2 and phot1 [55–58]) are short-lived upon light exposure, and problems with turnover might lead to wrong assessment of the available light. It was shown previously that ubK48R induction leads to PhyA accumulation [25]. In addition, ubK48R induction may not allow for adequate protection against photodamage. We know from previous experiments [24] that toxicity of ubK48R expression is lower under low light, and that ubK48R expressing plants accumulate reactive oxygen species. This accumulation occurs in a background of down-regulated ROS protective genes, a change that would be more appropriate for low light conditions, where photodamage as a major source of ROS is eliminated (see also Notes S2; for recent reviews on light stress, see [59, 60]). Regarding transcriptional changes of photoreception, differences were only found in phototropin-dependent blue light signalling. Derailment of this signalling may impact on light assessment, or on light protection.

### Suppressor mutations support the hypothesis that failure in a single process is the major cause of lethality

We had isolated EMS-induced mutations that suppress the phenotypic changes caused by ubK48R expression, called *sud* (**s**uppressor of **u**biquitin K48R induced cell **d**eath) mutations [24]. In this work, we used one of these mutants, *sud2–1*, for further studies, which resulted in identification of a T-DNA insertion mutant with *sud* phenotype. Approximately 40 genes that are significantly up-regulated upon ubK48R expression in the WT background are not up-regulated to the same extent in the *sud2–1* background (Table S6). We asked whether any of the induced genes are causally involved in seedling death. Among nine tested up-regulated genes (mostly of unknown function), a T-DNA insertion in At1g62290 (re-named SUD7) suppressed cell death (Fig. 3). SUD7 (also listed as putative aspartic protease A2, PASPA2) belongs to a family of saposin-like aspartate proteases and its induction depends on transcription factors FUS3 and LEC1 [61]. Asp proteases have previously been identified as membrane-associated components of several pathways [62, 63]. SUD7 is up-regulated by drought, by inhibition of chloroplasts [36, 46, 47], and under conditions where light exposure and chloroplast development are not in balance, e. g. in a quintuple phytochrome mutant, or in a quadruple phytochrome interacting factor mutant [43–45]. SUD7 could therefore be involved in removal of (light-damaged) chloroplasts, a process that overshoots when ubK48R is induced. Interestingly, a role for a protease in high light response was recently suggested [64]. Clearly, further investigations are necessary to clarify the role of SUD7 in cellular processes.

The NRL16 gene [16] co-segregates in crosses with the *sud2–1* allele, and is the only gene of the UPS linked to the SUD2 locus. A T-DNA insertion mutant was therefore tested for a potential *sud* phenotype. NRL16 is sequence-related to NPH3, a component of phototropin signalling. It has the characteristic NPH3 homology domain, a coiled-coil domain and a BTB domain that is present in subunits of cullin 3-based ubiquitin ligases. Two other NPH3 homologs, RPT2 and NCH1, were previously shown to operate in phototropin-mediated responses [13, 65]. NRL16 is in another clade, which is actually closer to NPH3 than the RPT2/NCH1 clade. NRL16 is highly expressed in mature seeds, and its expression increases during early seedling growth (Arabidopsis eFP browser http://bar.utoronto.ca/efp/cgi-bin/efpWeb.cgi). The nrl16 protein interacts with both PHOT2 and nph3 in a yeast two hybrid assay (Fig. 7). This Y2H interaction has yet to be confirmed by an independent interaction assay such as co-immunoprecipitation, and it should also be noted that a Y2H interaction of a phototropin with an nph3 family member does not automatically imply biological functionality (see ref. [66]). Nonetheless, the data are consistent with an nrl16 – phot2 signalling axis that requires protein turnover at the early seedling stage.

### A protective role for *PHOT2* in chloroplast development

The finding that a mutation in NRL16 acted as a suppressor, and transcriptional mis-regulation of phototropin inspired us to directly test for phototropin participation. If failure to inactivate a phototropin-containing complex via proteolysis is a cause for death, then plants lacking phototropins may be better off after ubK48R expression. Combination of a *phot1* mutation with the transgene was ineffective, or had a slightly negative influence on chloroplast development (Fig. 5). We generated mutations in *PHOT2* by CRISPR/Cas9 in the ubK48R transgenic background. Two frameshift mutations (*phot2–81* and *phot2–82*, a 1-bp and a 2-bp deletion in the LOV1 domain, respectively) were tested (Fig. 6). They resulted in a notable improvement of photomorphogenesis and seedling development. In particular, seedlings accumulated less anthocyanin and had more expanded cotyledons. A much higher fraction also formed leaf primordia. Nonetheless, most seedlings eventually stopped growth (Fig. 6).

A potential decrease-of-function *phot2* allele with in-frame deletion of nine base pairs (*phot2–83*) acted as a more efficient suppressor in our assay. Western blotting showed that the *phot2–83* allele resulted in a lower abundance of the protein compared to WT phot2 (Fig. 6). These suppressor results fit with the data that several components of phototropin-related light signalling display transcriptional changes. So far, the contribution of phytochromes and cryptochromes to seedling de-etiolation was clearly documented [67], whereas participation of PHOT2 in this process has not been demonstrated. However, a role for PHOT2 in protection from light damage by chloroplast re-positioning is established, and the single phytochrome of Chlamydomonas impacts on the transcriptional program to induce photo-protective genes [19], so that an additional photo-protective activity of PHOT2 manifested at the level of gene expression would be evolutionarily plausible. In our data set, a transcription factor of light stress protection (RHL41) is down-regulated.

**Fig. 9:**
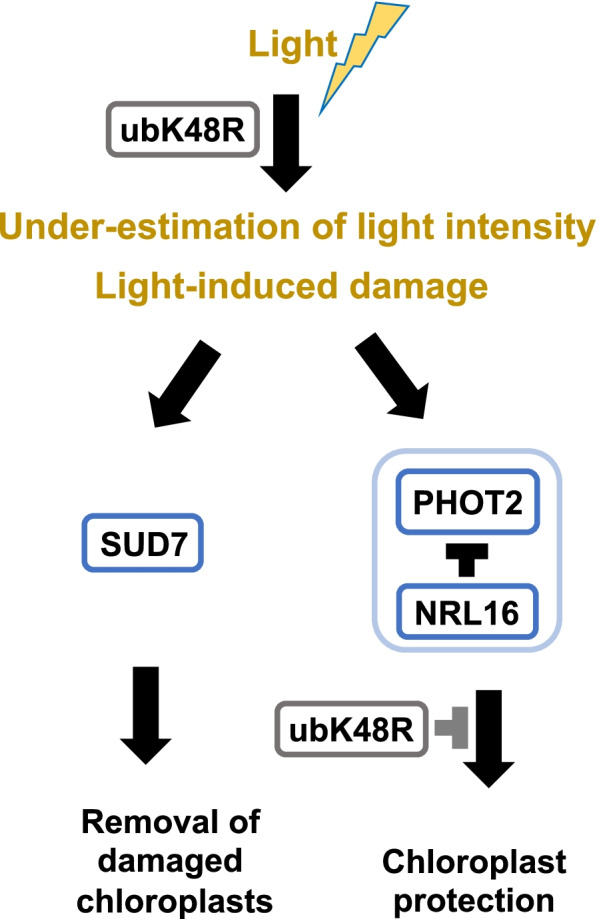
Model for interference of ubK48R expression with chloroplast de-etiolation. Upon ubK48R expression, the amount of available light during seedling de-etiolation is not correctly measured. This would not be lethal, if mechanisms of light protection were induced appropriately. High light intensity and/or emerging signals of light-induced damage are known to activate Phototropin 2 (PHOT2), leading to re-positioning of chloroplasts. In the model, a PHOT2-containing complex triggers, in addition, the expression of photo-protective genes. Light damage can also result in removal of damaged chloroplasts, which is supported by SUD7. Inhibition of proteolysis results in over-shooting of the chloroplast removal branch, whereas protective activity of PHOT2 is decreased. NRL16 is a natural inhibitor of this activity, so that its removal can partially compensate for a decrease in proteolytic activity. Likewise, certain mutations in PHOT2 decrease dependence on the proteolysis step, and mutations in SUD7 decrease chloroplast removal activities

In order to support or dismiss the hypothesis that misfunction of a phot2 containing complex compromises protection of chloroplasts from light damage, we analyzed chloroplast ultrastructure (Fig. 8). Induced RV86–5 seedlings showed severe deviation from normal chloroplast development (Fig. 8C, D), whereas seedlings with the *phot2–83* suppressor allele could transit development from etioplast to chloroplast (Fig. 8F). Most importantly, etioplast to chloroplast transition started in the induced RV86–5 line, as visible by appearance of thylakoids, but chloroplasts were actively disassembled, presumably by mechanisms that include autophagic processes [34, 68]. These findings are summarized in the model Fig. 9, where blue light perception by phot2 is a quantitative regulator of chloroplast protection from light damage. The model also sets the stage for future investigation of the indicated genes in genetic backgrounds other than RV86–5.

## Conclusions

Transcriptome and other omics data show that partial inhibition of protein turnover results in failure of chloroplast development in seedlings. The genetic data indicate that a pathway related to phot2 signalling is critically responsible. In our working model, a phot2 containing complex protects chloroplasts against photo-damage, but needs protein turnover to execute this activity.

## Methods

### Plant growth

Plants used in this work were *Arabidopsis thaliana* accession Col-0 (unless stated otherwise), obtained from Dr. Csaba Koncz (Max Planck Institute for Plant Breeding Research, Cologne Germany). Unless indicated otherwise, seedlings were grown on plates with Murashige and Skoog (MS) salts and vitamins, 1% sucrose, with 0.8% Phyto Agar or 0.3% Gelrite (all from Duchefa) in a 16 h light/8 h dark cycle at 23 °C under white fluorescent light. Plant propagation was under similar conditions in a controlled growth chamber environment. Supplements as indicated were: Hygromycin (Roche), 15 mg/l; dexamethasone (DEX), 0.7 μM (if not indicated otherwise); methotrexate, 0.15 mg/l. For growth in liquid culture, seedlings were germinated in 24 well microtiter plates containing liquid MS medium, exposed to 10 μM DEX and to 80 μM Zebularine. Amino acid supplementations and medium without nitrate are listed in Methods S1.

### RNA expression analysis

Total RNA was extracted and assayed in three biological replicates with three technical replicates each. For details, see Methods S2. All transcriptome data (CEL and CHP files) were submitted to a public repository database (http://www.ebi.ac.uk/microarray/), ArrayExpress accession number: E-MEXP-1454.

### Proteome analysis

Proteome analysis was carried out as described previously. The whole experiment was carried out in two complete biological replicates, each consisting of three biologically distinct samples pooled from 20 seedlings. For details, see Methods S3. The mass spectrometry proteomics data have been deposited to the ProteomeXchange Consortium via the PRIDE (https://www.ebi.ac.uk/pride/ [69];) partner repository with the dataset identifier PXD012815.

### Metabolite extraction and analysis

Polar metabolites were extracted as described previously [70] with few modifications as indicated in Methods S4. At least 50 seedlings were collected per replicate and the whole experiment was carried out in at least four biologically distinct replicates.

### Western blotting

Proteins from seedlings were extracted from pulverized frozen tissue and processed as described in Methods S5.

### Vector constructions

For yeast two hybrid vectors, open reading frames were PCR amplified and inserted into SmaI digested pGADT7 (for activation domain fusions) and pGBKT7 (for DNA binding domain fusions), respectively, via In-Fusion Cloning (TaKaRa). Oligonucleotides used are listed in Table S7. For construction of Cas9 vectors [71], an sgRNA for *PHOT2* exon 2 was selected via Zhang Lab Guide Design Tool (https://zlab.bio/guide-design-resources; 5′_ACTCCTATCAAGGACGACCA(GGG)_3’) and cloned as described in Methods S6.

### Electron microscopy

Ten-day old seedlings exposed 7 days to 0.7 mM DEX were fixed using 2% glutaraldehyde and 2% paraformaldehyde in 0.1 M Sodium Cacodylate Buffer (NaCB). Seedlings were kept in a desiccator for 2 h and o/n rotation at room temperature. Washings were performed with 0.1 M NaCB, followed by subsequent steps: staining with 1% Osmium tetroxide in 0.1 M NaCB on ice for 40 min, two rinsing steps in NaCB, one rinsing step in ddH_2_O. Samples were dehydrated in a graded series of acetone (40, 60, 80 and 100%) on ice, and embedded in Agar 100 epoxy resin. Seventy nm sections were collected with 100 mesh Cu/Pd grids (Agar Scientific) and post-stained with 2% uranyl acetate and Reynolds lead citrate. Sections were examined with an FEI Morgagni 268D (FEI, Eindhoven, The Netherlands) at 80 kV. Images were acquired using an 11 megapixel Morada CCD camera (Olympus-SIS).

### Analysis of GO terms

GO terms were analysed via the tools provided by TAIR (arabidopsis.org), via the PANTHER classification system.

### Public resources

The following cDNAs from RIKEN [72, 73] were used to amplify open reading frames: *PHOT2* (AT5G58140.1): pdx62360; NRL16 (AT3G44820.1): pdz08478; NPH3 (AT5G64330.1): pdx62834. Arabidopsis T-DNA insertion lines were from NASC: lines SALK_040283 and SALK_143045 (*nrl16–1* and *− 2*, respectively) [74], and *sud7* (At1g62290) line FLAG_554E08 [75].

### Accession numbers

SUD7 (**S**UPPRESSOR OF **U**BK48R-INDUCED CELL **D**EATH 7; also annotated as putative aspartic protease A2, PASPA2), At1g62290; NRL16, At3g44820; *PHOT2*, At5g58140; NPH3, At5g64330.

## Supplementary Information

**Additional file 1.**

## Data Availability

The data generated and analyzed during this study are included in the article and its supplementary information. Mutant lines are available upon request from the corresponding author.
