# Supplementary material for: Transcriptome, metabolome and suppressor analysis reveal an essential role for the ubiquitin-proteasome system in seedling chloroplast development
Source: BMC Plant Biol. 2022 Apr 8;22:183. doi: 10.1186/s12870-022-03536-6 (PMC8991883; doi:10.1186/s12870-022-03536-6)
Supplement: Supplementary file 1 — Additional file 1. [file 12870_2022_3536_MOESM1_ESM.zip › Supplementary Text and Figures_12.pdf]

Supplementary Text and Figures to:

Transcriptome, metabolome and suppressor analysis reveal an essential role for the ubiquitin-proteasome system in seedling chloroplast development

Prabhavathi Talloji, Lilian Nehlin, Bruno Hüttel, Nikola Winter, Martin Černý, Hana Dufková, Bulut Hamali, Katarzyna Hanczaryk, Jan Novak, Monika Hermanns, Nicole Drexler, Karolin Eifler, Nikolaus Schlaich, Břetislav Brzobohatý, Andreas Bachmair

Supplementary information consists of:

Supplementary Text and Figures:

Notes S1 Plant death and growth arrest after induction of ubK48R are not mitigated by additional amino acid supply.

Notes S2 Additional changes in transcription after ubK48R expression.

Notes S3 Proteome changes after ubK48R induction.

Notes S4 Growth arrest and death upon ubK48R expression with the GVG inducible system are not a consequence of high GVG levels.

Notes S5 Supplementary references.

Methods S1 Plant growth media.

Methods S2 RNA expression analysis.

Methods S3 Proteome analysis.

Methods S4 Metabolite extraction and analysis.

Methods S5 Western blotting.

Methods S6 Vector constructions.

Methods S7 Lugol staining.

Figure S1. Growth of line RV86-5 on plates containing DEX is not improved by the presence of amino acids.

Figure S2. Arabidopsis seedlings can grow with amino acids as sole nitrogen source.

Figure S3 Growth habit of plants discussed in this work.

Figure S4 Western blot after induction of the ubK48R transgene by DEX in different genetic backgrounds

Figure S5 Lugol staining of leaves to assess starch content

Figure S6 Growth of line RV86-5 is not impeded by its level of the GVG transcriptional activator.

Figure S7 Whole size Western blot of Figure 4.

Figure S8 Whole size Western blot of Figure 6C.

Figure S9 Whole filters of Western blot shown in Figure S4.

Figure S10 Whole image of agarose gel with fragments from RT-PCR reaction shown in Figure S6A

Additional file: Table S1 Genes induced by ubK48R induction (Excel file).

Additional file: Table S2 Genes repressed by ubK48R induction (Excel file).

Additional file: Table S3 Enriched terms of GO biological process terms (PDF file).

Additional file: Table S4 Proteomics data of ubK48R plants (Excel file).

Additional file: Table S5 Metabolome changes by ubK48R induction (Excel file).

Additional file: Table S6 Transcript differences between RV86-1 and RV86-1 *sud2-1* upon induction (Excel file).

Additional file: Table S7 Oligonucleotides used in this work (PDF file

SUPPLEMENTARY TEXT:

Notes S1 – Plant death and growth arrest after induction of ubK48R are not mitigated by additional amino acid supply

Inhibition of the UPS has lethal consequences in all eukaryotes tested so far. In most cases, exposure to proteasome inhibitors such as MG132 is used to block degradation of ubiquitylated proteins (refs. [26-28] of the main text). Bertolotti and coworkers found that, whereas cessation of cell division after inhibition of the UPS cannot be avoided, a concomitant cell death does not occur in yeast and mammalian cells if extra amino acids are added to the culture medium (ref. [41] of the main text). We therefore set out to investigate whether expression of ubiquitin variant ubK48R to inhibit the UPS (ubiquitin proteasome system) in seedlings does also result in seedling lethality due to amino acid shortage. We tested whether addition of amino acids to growth media allows better growth or even survival of induced RV86-5 plant seedlings. We placed seeds in parallel on plates containing DEX with either a mixture of amino acids (see Methods S1 for concentrations), or no addition. Figure S1 shows that no difference in growth was observed (Figure S1, A vs. B). Fourteen randomly chosen seedlings were placed on medium without DEX to assess whether seedlings from plates with additional amino acids could resume growth. However, this was not the case (Figure S1, C vs. D). Only two seedlings from both media were able to resume growth (examples Figure S1, E vs. F). The recovering plants had a green apical meristem region, but had been growth-arrested on the DEX medium. This suggests that resumption of growth after transfer to medium lacking DEX is possible for plants that had not already died. Plants can take up amino acids from the medium (Rentsch et al. 2009; Tageder and Ward 2012), but the experiments of Figure S1 do not rule out that amino acid uptake is insufficient to significantly influence plant growth. Therefore, a different form of amino acid shortage, caused by complete lack of nitrogen supply, was used and compared to growth with amino acid supplementation. Figure S2 shows three weeks-old seedlings, and those with amino acid supply are viable and grow (Figure S2B vs. A), although growth lags behind growth on medium supplemented with standard nitrogen source (see Methods S1). This experiment indicates that amino acid uptake into Arabidopsis seedlings is sufficient to promote a moderate rate of growth and to mitigate consequences of amino acid shortage. A tentative conclusion of these experiments is therefore that, in contrast to animals or fungi, inhibition of the UPS has a lethal effect in plants at an inhibitory level that is not (yet) generating amino acid shortage. Alternative interpretations are that a generally high level of vacuolar protein

turnover in plants provides a sufficient supply of free amino acids even when the UPS is significantly inhibited, or that down-regulation of protein synthesis (see below) sufficiently compensates for the lack of protein re-cycling.

Notes S2 – Additional changes in transcription after ubK48R expression

Transcriptional changes in ubiquitylation and ubiquitin-dependent protein turnover – Somewhat surprising, there was no net up-regulation of components of ubiquitin-dependent protein turnover in response to 24 hr DEX exposure. 22 genes were 2.4-fold or more down-regulated, 20 were up-regulated 2.4-fold or more. Most of the changes were in subunits of cullin type ligases, none of them was in a proteasome subunit. Thus, the perturbed plants seemed not to rely on the UPS to counter-balance the life-threatening changes that happen as a consequence of ubK48R induction.

Protein phosphorylation and dephosphorylation – 36 kinases are up-regulated more than 2.4 fold, and 23 kinases are down-regulated more than 2.4 fold. 10 of the up-regulated, and 12 of the down-regulated kinases contain an LRR domain. One kinase interacting protein and one kinase inhibitor are up-regulated. Furthermore, 6 phosphatases are up-regulated and 7 phosphatases are down-regulated. In sum, there are more kinases induced than down-regulated. This is particularly obvious if LRR kinases are omitted from the count (26 up- versus 11 down-regulated). Kinases with decreased transcription are mostly receptor type kinases (with extracellular domains other than LRR). Plants thus rely more on changes in phosphorylation to cope with impaired protein turnover than on changes in ubiquitylation.

Plant hormonal pathways – Cessation of plant growth coincides with changes in plant hormone synthesis, distribution or response.

Auxin: Five enzymes of auxin biosynthesis are down-regulated, but only 1 is up-regulated. One enzyme of auxin desensitization is up-regulated. One auxin influx carrier (LAX3) is down-regulated, one up-regulated. Two efflux carriers (PIN1, PIN4) are up-regulated. Eight auxin-responsive transcriptional regulators are down-regulated, four are up-regulated. 6 (auxin-induced) SAUR genes are down-regulated, but only 2 are up-regulated. These changes may result in a net decrease in intracellular auxin concentration and auxin response.

Cytokinin: Two enzymes of cytokinin biosynthesis are down-regulated, one enzyme of cytokinin turnover is up-regulated. Three cytokinin-responsive transcription factors are down-regulated, one is up-regulated. Four genes encoding response regulators (ARR proteins) are down-regulated. We conclude that there may be a decrease in cytokinin

biosynthesis and response, which his consistent with the role of cytokinin in chloroplast development (Lochmanová et al. 2008; Hlousková et al. 2019).

Brassinosteroids: Four genes of BR response are down-regulated (including transcription factors BEE2 and BEE3). One enzyme with predicted involvement in BR biosynthesis is down-regulated. Two genes of BR response are up-regulated, one of them is listed as both BR and cytokinin-regulated. This suggests that brassinosteroids do not give extra growth stimulation to induced plants, or may even contribute to the observed cessation of growth.

Gibberellin: One gene involved in (general) steroid biosynthesis, and two genes of GA biosynthesis are up-regulated. No GA biosynthesis gene is down-regulated more than 2.4 fold. Likewise, three GA responsive genes are up-regulated, no gene regulated exclusively by GA is down-regulated. GA is therefore the only growth-promoting hormone with a positive correlation to induction of the ubiquitin variant. Interestingly, GA can be linked to cell length increases, stimulating outgrowth of seedlings to reach zones of better light supply. In sum, however, changes in hormone metabolism and response are consistent with the observed decrease in growth that results from decreased performance of chloroplasts.

Defense-related pathways – 47 genes with potential connection to defense responses are down-regulated, 44 are up-regulated. There are more JA-induced genes up- than down-regulated: 12 up-regulated versus 7 down-regulated. Moreover, one of the down-regulated genes is WRKY70, a suppressor of JA responses. This change thus has the effect of up-regulating JA responses. Likewise, three enzymes of ethylene biosynthesis are down-regulated, but three up-regulated. Many ethylene-responsive genes are also JA-responsive. Among ethylene-responsive genes that are not annotated as JA responsive, 6 are up-regulated and four are down-regulated. Thus, there is apparently a slight net-increase in JA / ethylene responses. 5 genes annotated as SA responsive are down-regulated, and 7 are up-regulated. The general impression is therefore that the minor changes in defense-related genes are governed more by JA and ethylene, than by SA. Biosynthesis of defense-related isoflavonoid compounds and of UV protecting flavin compounds is down-regulated. This is consistent with the hypothesis that plants "think" to be in low light, so that no additional UV protection is necessary. 2 genes of flavonoid biosynthesis are more than 10-fold down, 7 genes between 10- and 2.4 fold. Only one anthocyan modifying enzyme is up-regulated, and one transcription factor, anthocyanless 2, is mildly up-regulated (2.3-fold). Nonetheless, there is an up-regulation of early phenylpropanoid pathway genes such as PAL, leading to the conclusion that lignin biosynthesis is up, but not biosynthesis of protective flavin compounds (see below, cell wall remodelling). The lack of clear net induction of defense genes also

supports our previous conclusion that ubK48R expression, and not high GVG transcription factor levels, are the cause for the major changes we observe in induced RV86-5 plants, because high GVG abundance is correlated with induction of defense genes such as PR proteins (Kang et al. 1999).

Detoxification pathways (efflux carrier, heavy metal induced genes) – There is no indication that inhibition of ubiquitin-dependent proteolysis is interpreted as intoxication by xenobiotics (some of which may lead to accumulation of denatured proteins). 12 MatE, EamA or ABC transporters that could potentially extrude xenobiotics are down-regulated, whereas only 7 are up-regulated. This contrasts with genes that counteract heavy metal intoxication: proteins that export heavy metals or are involved in synthesis of heavy metal ligands such as Nicotianamine are up-regulated (12 genes), and only 2 genes of this class are down-regulated. This may relate to heavy metals as protein denaturants. Partial inhibition of ubiquitin-dependent proteolysis may therefore lead to a mild increase in the abundance of denatured proteins, which is interpreted by the plant as heavy metal intoxication. In order to address the question why plants with inhibited ubiquitin conjugation die, we specifically looked for mis-expression of genes previously associated with cell death processes. 7 genes with annotation as senescence-induced or -related are down-regulated, and 6 genes of this class are up-regulated. Among the down-regulated genes are ORE1, SEN1, and WRKY22, whereas ORE7 and WRKY45 are among the up-regulated genes. Regarding genes with potential connection to fast cell death programs, we counted 4 down-regulated and four up-regulated genes. Interestingly, two of the down-regulated genes presumably act in a pro-apoptotic fashion in defense responses (DND1 and DND2), and one of the up-regulated genes is a member of the BAX inhibitor family, which may actually raise the cell death threshold. A cysteine protease with role in xylem differentiation (proto-xylem cell death) is up-regulated, whereas another member of this class is down-regulated. Metacaspase 1 (AMC1) is 2.5-fold up-regulated. Taken together, these latter transcriptional changes do not uncover an obvious trigger of fast cell death, which is consistent with the hypothesis that cell death occurs because of mis-regulated chloroplast development/protection.

Red-ox homeostasis in the cytoplasm – We have previously shown that cell death associated with ubK48R expression coincides with increased intracellular presence of reactive oxygen species (Schlögelhofer et al. 2006). It was therefore of interest to see whether DEX-exposed plants strengthen their enzyme machinery dealing with ROS. Surprisingly, this was not the case. Our favourite interpretation is that when light exposure of chloroplasts is no longer a source of ROS to be taken care of (without light, photo-oxidative processes would indeed be

no source of oxidative stress), ROS protection is down-regulated. A decreased synthesis of red-ox homeostasis enzymes may therefore be the result of co-regulation with chloroplast genes. 40 genes that potentially contribute to red-ox homeostasis were down-regulated, but only 16 were up-regulated (chloroplast and mitochondrial redox regulators were included in the section devoted to these organelles, and are not counted here). We included in this group thioredoxins and glutathione S transferases, oxidoreductases with Rossmann fold, and Fe- and 2-oxoglutarate-dependent redox enzymes. The net down-regulation in this group may be compared to the distribution of changes for cytochrome P450 type enzymes, which are involved in multiple biosynthetic redox reactions, but are not known to contribute to ROS protection. There were 11 up- and 11 down-regulated P450 enzymes.

Cell wall remodeling – Three genes of cell wall metabolism are down-regulated more than 10-fold, 28 genes between 10- and 2.4-fold. 6 genes of cell wall metabolism are up-regulated more than 10-fold, 33 genes between 10- and 2.4-fold, and six between 2.4- to 2.2-fold. Among the down-regulated genes are inhibitors of cell wall modifying enzymes, among the up-regulated genes enzymes of cell wall modification (e.g., pectin esterases). Key genes of lignin biosynthesis are highly up-regulated (e.g., PAL4 is up-regulated 14-fold). These data indicate that cell walls are remodeled, most likely strengthened, upon ubK48R induction. Because there is no net up-regulation of defense-related genes, the re-modeling may not be part of a defense response. Likewise, cell wall biosynthesis has no obvious connection to chloroplast malfunction, so that our current hypothesis is that cell wall integrity and/or strengthening of the cell wall is regulated by a circuitry that depends on ubiquitin-mediated protein turnover, but that is independent of chloroplast development and protection.

Notes S3 – Proteome changes after ubK48R induction

Two-week old seedlings were exposed to 0.7 µM dexamethasone (see Methods S3). An additional experiment applied ten times higher DEX concentrations (7 µM DEX). A total of 3220 proteins, representing over 1800 protein groups, were identified by stringent criteria. A set of 45 proteins showed significant and reproducible changes after 24 hr 0.7 µM DEX treatment. 26 of these also displayed transcriptional changes, which were with one exception in the same direction as the protein abundance changes. A further set of 50 proteins showed significant changes in abundance only after the higher (7 µM) DEX treatment. Of these, 32 were also among the differentially transcribed gene data set. For more details, see Table S5. Among all 95 proteins with significant abundance changes, 31 were from chloroplasts and were reduced in abundance, only eight were more abundant. Eight proteins associated with

biogenesis of cytoplasmic ribosomes were reduced in abundance, four chaperone proteins were less abundant, and three proteins with link to red-ox homeostasis were less abundant. Although there is little overlap between down-regulated proteins and the genes found to be transcriptionally down-regulated, the data confirm and extend the transcriptome data by indicating down-regulation of chloroplast proteins. The fact that 61 of the 95 proteins with significant changes displayed lower abundance suggested that protein turnover is not completely blocked by the experimental setup (note that, for instance, autophagy should be unperturbed). Furthermore, none of 17 identified proteasome subunits displayed changed abundance, in agreement with the relative transcriptional non-responsiveness of the UPS to the perturbation (see above).

Notes S4 – Growth arrest and death upon ubK48R expression with the GVG inducible system are not a consequence of high GVG levels

The induction system via vector pTA7002 (Aoyama and Chua 1997) was reported to have side effects by inducing off-target genes in quantitative dependence on the level of transcription factor GVG (Kang et al. 1999). We therefore monitored genes induced by the GVG transcription factor acc. to Kang et al. (1999). For instance, PR-2 (At3g57260) is inducible by high GVG levels, but not induced in line RV86-5. Similarly, PR-1 (At2g19990) is down-regulated by high GVG levels, but not significantly down-regulated in line RV86-5. We tentatively concluded that GVG levels in line RV86-5 are not excessively high. Furthermore, we compared line RV86-5 with two other lines that express different proteins via GVG induction system. Firstly, line cK2 expresses an activated protein kinase. Transcriptional changes following kinase induction were published (Marques-Bueno et al. 2011), and these have little in common with the changes following induction of ubK48R in line RV86-5. Secondly, a line expressing *E. coli* β-glucuronidase under control of the GVG induction system was used for comparison. Figure S6A shows an RT-PCR experiment to compare the levels of transcription factor GVG in these three lines, because Kang et al. (1999) had reported developmental defects and expression of defense genes with plants that had high GVG expression levels. GVG is under control of the constitutive 35S promoter, and expression levels differ in different transgenic lines. We find that the expression of GVG is slightly higher in the cK2 line than in the RV86-5 line. The fact that these two lines have a distinctly different spectrum of transcriptional changes upon DEX induction supports the notion that changes are caused by the induced transgene, and not by off-target effects of

GVG. Likewise, the control line pTA-GUS13, expressing an inducible GUS reporter, has a level of GVG that almost matches that of RV86-5, but plants grow unimpeded after induction (Figure S6B). We therefore concluded that growth inhibition and death upon DEX application to line RV86-5 are a consequence of the induced ubiquitin variant, and not of excess GVG expression.

Notes S5 – Supplementary references

Hloušková P, Černý M, Kořínková N, Luklová M, Minguet EG, Brzobohatý B, Galuszka P, Bergougnoux V. Affinity chromatography revealed 14-3-3 interactome of tomato (Solanum lycopersicum L.) during blue light-induced de-etiolation. J Proteomics. 2019;193:44–61.

Lochmanová G, Zdrahal Z, Konecná H, Koukalová S, Malbeck J, Soucek P, Válková M, Kiran NS, Brzobohatý B. Cytokinin-induced photomorphogenesis in dark-grown Arabidopsis: a proteomic analysis. J Exp Bot. 2008;13:3705–3719.

Rentsch D, Schmidt S, Tegeder M Transporters for uptake and allocation of organic nitrogen compounds in plants. FEBS Lett. 2007;581:2281–2289.

Tageder M, Ward JM. Molecular evolution of plant AAP and LHT amino acid transporters. Front Plant Sci. 2012;3:21.

Methods S1 – Plant growth media

Amino acids as supplement to plant media were added from a filter-sterilized stock solution (His 200 mg/100 ml, Met 200 mg/100 ml, Trp 300, Thr 1500, Leu 800, Lys 400, Phe 500, Tyr 200, Ile 200, Val 650, Arg 300, Asp 1000) after autoclaving. The final concentrations were: 20 mg/l His, 20 mg/l Met, 30 mg/l Trp, 150 mg/l Thr, 80 mg/l Leu, 40 mg/l Lys, 20 mg/l Tyr, 20 mg/l Ile, 65 mg/l Val, 30 mg/l Arg, 100 mg/l Asp.

Medium without nitrogen source was 0.61 g/l Murashige and Skoog modified Basal Salt mixture without nitrogen, phosphorous and potassium (Phyto Technology Laboratories M407) with 0.17 g/l KH_2PO_4, 10g/l sucrose, 100 mg/l *myo*-Inositol, 20 mg/l Thiamine, 1 mg/l Nicotinic acid, 1 mg/l Pyridoxin, 0.2 mg/l Biotin and 0.35 % Phytagel), was either left un-supplemented, supplemented with amino acids (20 mg/l His, 20 mg/l Met, 30 mg/l Trp, 150 mg/l Thr, 80 mg/l Leu, 40 mg/l Lys, 20 mg/l Tyr, 20 mg/l Ile, 65 mg/l Val, 30 mg/l Arg, 100 mg/l Asp), or supplemented with NH_4NO_3 (0.82 g/l) and KNO_3 (0.95 g/l) to restore standard MS media conditions.

Methods S2 – RNA expression analysis

Total RNA was extracted from rosette leaves (>1 cm in length) using an RNeasy mini kit (Qiagen, Hilden, Germany). Transcriptomes were analysed using 1µg of total RNA as starting material. Targets were prepared with the one-cycle cDNA synthesis kit followed by biotin-labelling with an IVT labelling kit (MessageAmpII-Biotin Enhanced kit (Ambion) and hybridized for 16 h as recommended by the supplier (Gene expression analysis manual, Affymetrix).

Methods S3 – Proteome analysis

Seedlings were cultivated for 15 days (21 °C/19 °C day/night temperatures, with a 16 h photoperiod; 90 µmol m^{-2} s^{-1} light intensity) on a textile mesh (Uhelon 120T Silk & Progress, Czech Republic) placed on top of 1% (w/v) agar medium containing half strength Murashige and Skoog medium. 24 h prior the leaf sampling, the meshes with seedlings were transferred onto solid medium supplemented with (i) 5×10−4% (v/v) DMSO (mock buffer); (ii) 0.7 or 7.0 µM DEX in DMSO (final concentration, as for the mock).

The leaf tissue of 20 Arabidopsis seedlings per replicate was collected and homogenized in liquid nitrogen. The material was extracted by acetone/TCA and phenol extraction, then digested in solution with immobilized trypsin beads (Promega, http://www.promega.com/). The resulting peptides were desalted, dried and dissolved in 0.5% (v/v) formic acid in 5% (v/v) acetonitrile, then analyzed by nanoflow C18 reverse-phase liquid chromatography using a 15 cm Ascentis Express Column (0.1 mm inner diameter; Sigma-Aldrich) and a Dionex Ultimate 3000 RSLC nano UPLC system (Thermo, www.thermoscientific.com) directly coupled to a CaptiveSpray nanoESI source (Bruker) and a UHR maXis impact q-TOF mass spectrometer (Bruker, www.bruker.com). Peptides were eluted with a 180-min, 4% to 40% acetonitrile gradient. MS/MS spectra were acquired in an intensity-dependent mode at a rate of 2–20 Hz with a maximum of 20 precursor ions and an MS spectra rate of 2 Hz.

Raw files obtained from the MS analysis were analyzed by Profile Analysis 2.1 (Bruker) and MS precursors with significant differences in two biological replicates (absolute ratio ≥1.4, with t-test p-values <0.05), and peptide spectra were searched against the TAIR10 Arabidopsis database using the Mascot 2.4 (database search criteria: trypsin; variable modifications – methionine oxidation, NQ deamidation, ST phosphorylation; peptide tolerance – 10 ppm; allowed one missed cleavage; MS/MS tolerance – 0.06 Da) and Bruker's ProteinScape percolator algorithms (target FDR < 1%) to identify source proteins (using high-confidence peptides, $p < 0.05$ with at least one distinct proteotypic peptide per protein).

Quantitative differences were further manually validated by comparing corresponding peptide ion signal peak areas in Skyline 1.4 (MacCossLab Software; https://skyline.gs.washington.edu). To complement our results, three representative raw files per biological replicate were recalibrated and searched against Araport11 database using ProteomeDiscoverer 2.2 and Sequest algorithm (database search criteria: trypsin; 35 ppm precursor mass tolerance; 0.1 Da fragment mass tolerance; allowed one missed cleavage; dynamic modifications – oxidation M, deamidation N, Q and N-terminal acetylation. The mass spectrometry proteomics data have been deposited to the ProteomeXchange Consortium via the PRIDE partner repository with the dataset identifier PXD012815.

Methods S4 – Metabolite extraction and analysis
Seven-day-old seedlings were cultivated as described above on medium supplemented with DEX (0-10000 nM). Seedlings were collected, flash-frozen and homogenized in liquid nitrogen. Aliquots corresponding to ca 150 mg were extracted with 1 ml methanol/methyl tert-butyl ether/water (1:3:1) overnight. The resulting polar phase was separated by adding 0.5 ml of methanol:water (1:3), and 50 µL aliquots of the polar phase were concentrated on a SpeedVac (Thermo). Samples were derivatized by 20 µL of methoximation solution (40 mg methoxyamine hydrochloride in 1 mL pyridine) and incubated for 90 minutes at 30 °C with continuous shaking. After the incubation, 80 µL of silylation solution (N-methyl-N-trimethylsilyl trifluoroacetamide, MSTFA) was added and the mixture was incubated for 30 min at 37 °C with continuous shaking. GC-MS measurements were carried out on Q Exactive GC Orbitrap GC-MS/MS (Thermo Fisher) using Trace 1300 Gas chromatograph (Thermo Fisher). Samples were injected using the split mode (1:10, total volume 1µl at 250 °C) and ionized using the electron ionization mode. The mass spectrometer operated in the full scan mode, 60,000 resolution, scan range 50-600 m/z. Data were analysed by TraceFinder and CompoundDiscoverer (Thermo Fisher) using default workflow for GC-MS data. Skyline was used for the final quantification.

Methods S5 – Western Blotting
Proteins from seedlings were extracted from pulverized frozen tissue (TissueLyser II, Qiagen) by boiling 6 min at 95°C in extraction buffer (90mM HEPES pH 7.4, 2% SDS, 20mM DTT, 20µg/ml pepstatin (Roche, 10 253 286 001), and 10µl/ml Protease Inhibitor Cocktail (Sigma, P9599)) and subsequent centrifugation at 15.000xg for 10 min at room temperature. Supernatants were mixed with 2x sample buffer (final concentration: 50mM

Tris pH 6.8, 2% SDS, 10% glycerol, 1% beta-mercaptoethanol, 12.5mM EDTA 0.02% Bromphenol blue). For detection of POTOTROPIN2, a polyclonal antibody from rabbit (Agrisera AS10 721) was used 1:2000 in combination with a secondary anti rabbit IgG-HRP antibody (GE Healthcare NA934) 1:10000 in 5% milk PBS-T (0,05% Tween-20). DHFR was detected analogously with a home-made affinity-purified anti mouse DHFR antibody from rabbit 1:250 in combination with the secondary anti rabbit IgG antibody 1:5000, or with commercial anti-human DHFR antibody (Santa Cruz sc-377091). The signal was developed with Advansta WesternBright Sirius reagent and detected via a ChemiDoc™ Imaging System (BioRad).

Methods S6 – Vector constructions

Oligonucleotides for Yeast two hybrid vectors were: 2447, 2448 (PHOT2, AD); 2449, 2450 (PHOT2, DBD); 2435, 2436 (NRL16, AD); 2433, 2434 (NRL16, DBD); 2445, 2446 (NPH3, AD); 2431, 2432 (NPH3, DBD). Sequences are listed in Table S7.

The sgRNA sequence for *PHOT2* gene editing was cloned into pCHIMERA (Fauser et al. 2014) using site-directed mutagenesis and oligos 1751+1752. The fragment of the customized PHOT2-pCHIMERA vector containing the AtU6-26 promoter and the PHOT2 sgRNA was amplified with Q5 DNA polymerase (New England Biolabs) and transferred into the AvrII site of the pCas9-TPC vector (ref. [71] of the main text) by Gilson assembly (NEBuilder; New England Biolabs). *Agrobacterium tumefaciences* GV3101::pMP90::pSoup was transformed with the final vector LB::PcUb4-2i::Cas9wt::pea3A-ter::AtU6-26 prom::sgRNAPHOT2::term::bar resistance cassette::RB by electroporation and selected with 50mg/L spectinomycin, 50mg/L rifampicin, 5 mg/L tetracyclin, and 25mg/L gentamycin. Transformation in Arabidopsis RV86-5 line was performed by floral dipping. Seedlings (T0) were selected by spraying 20mg/L BASTA. Resistant plants were selected and genotyped for the presence of *bar* and *Ubi4-Cas9*. The PHOT2 fragment was amplified with primers 1643 and 1644 (Table S7) and assayed by Sanger sequencing. Selected plants were back-crossed to Col-0 to generate homozygous lines without Cas9 and Basta resistance.

Methods S7 – Lugol staining of seedlings

Seven-day old seedlings grown on plates with and without DEX were cleared of chlorophyll by dehydration steps with 30%, 50%, and 70% ethanol for 48 hrs and stained for 30 minutes with iodine-potassium iodide solution (Lugol´s solution, ROTH N052.1).

SUPPLEMENTARY FIGURES:

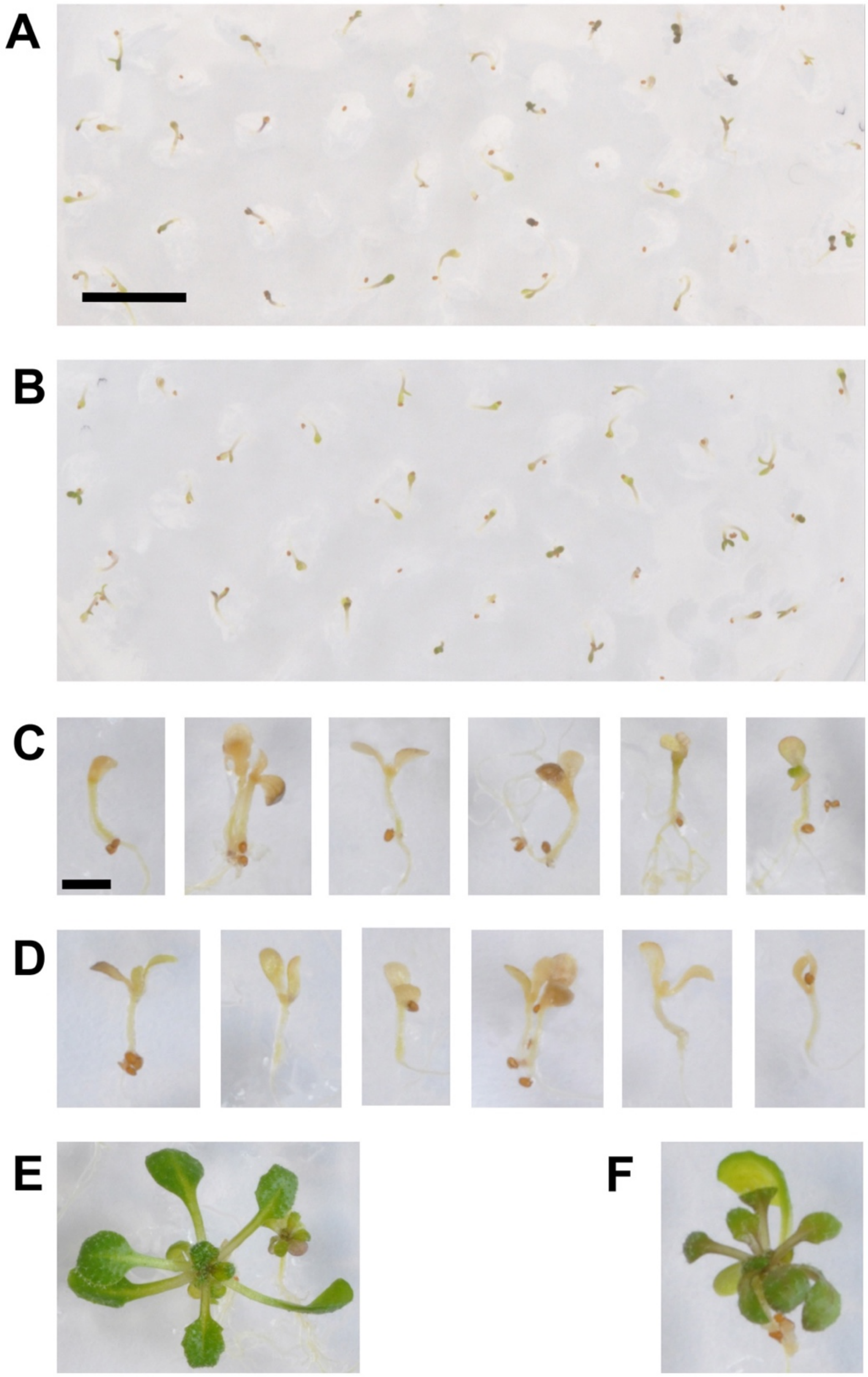

Figure S1. Growth of line RV86-5 on plates containing DEX is not improved by the presence of amino acids (**(A)**, with amino acids vs. **(B)**, without amino acids after ten days of growth). Plants from both growth conditions were put on plates without DEX for recovery. Plantlets from plates with amino acids **(C)** did not recover better than those from plates without amino acids **(D)**. In contrast, plants that retained their green color on either medium did resume growth in absence of DEX (**(E)**, plant from medium with amino acids; **(F)**, plant

from medium without amino acids). Recovery of plants in **(C)** to **(F)** was for three weeks. **(A)** and **(B)** (scale bar 1 cm), and **(C)** and **(D)** (scale bar 0.2 cm), respectively, are each at the same scale for size comparison.

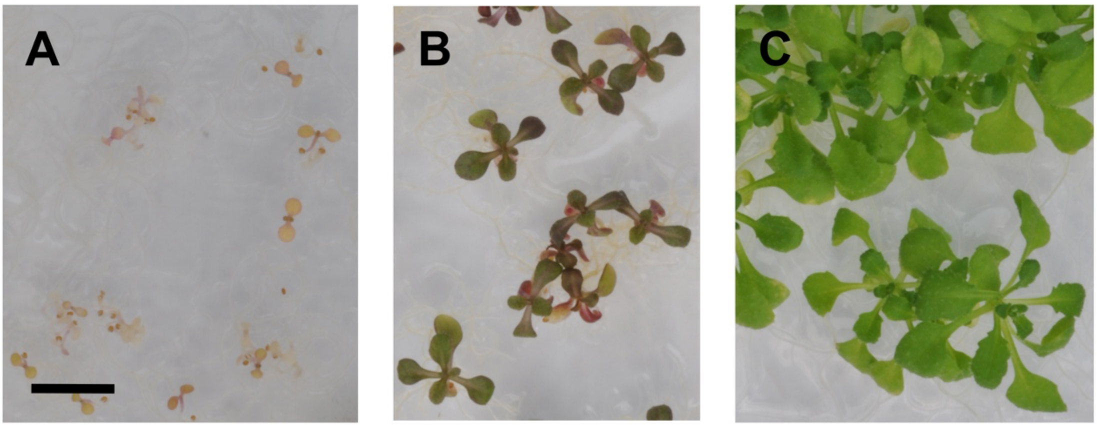

Figure S2. Arabidopsis seedlings can grow with amino acids as sole nitrogen source. Growth of line RV86-5 on plates containing no nitrogen source **(A)**, amino acids as sole nitrogen source **(B)**, or normal MS salt mixture **(C)**, all supplemented with 1% sucrose. Bar, valid for all three images: 10 mm. Image was taken three weeks after sowing.

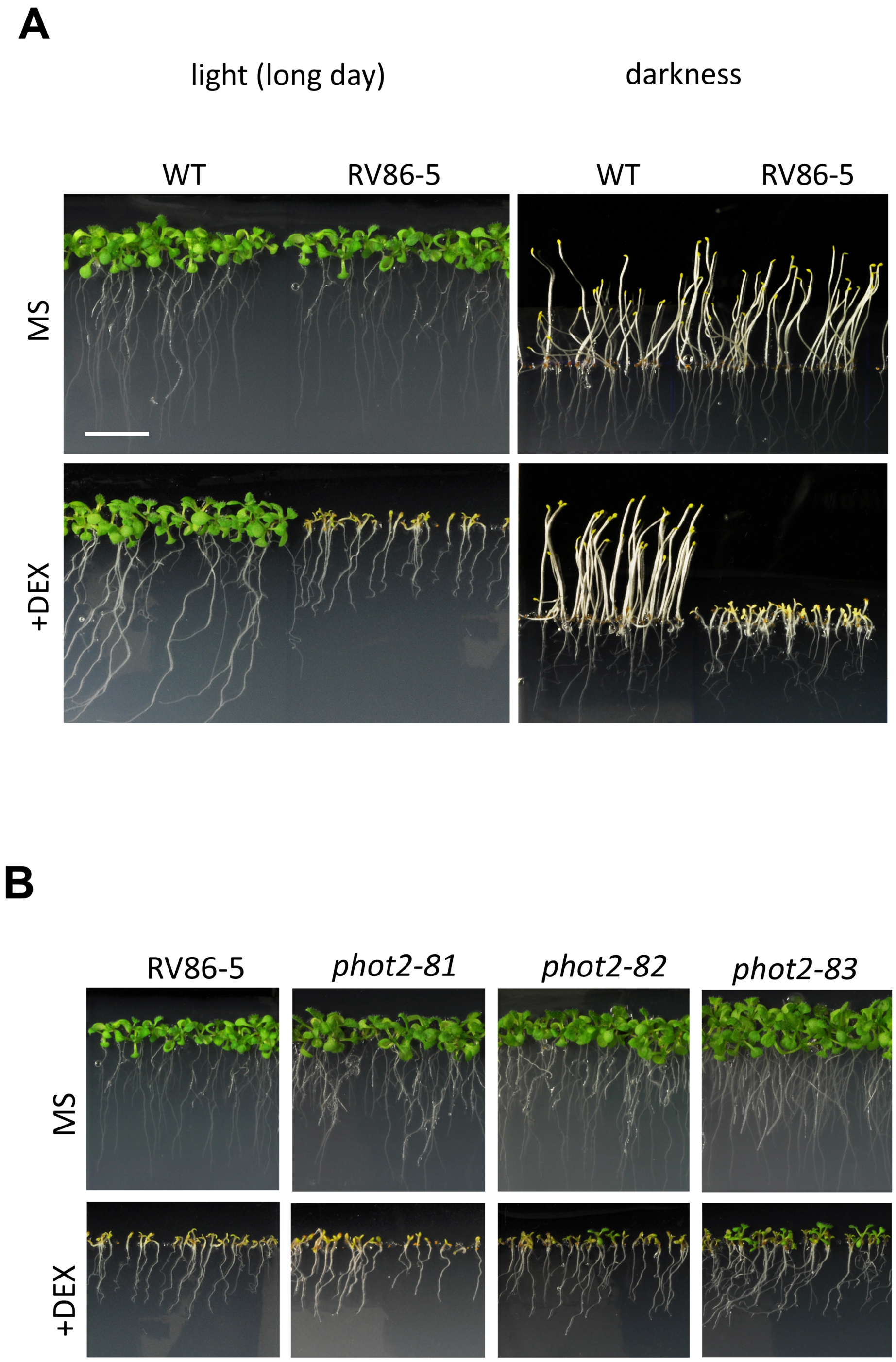
A
light (long day)
darkness
WT
RV86-5
WT
RV86-5
MS
+DEX
B
RV86-5
phot2-81
phot2-82
phot2-83
MS
+DEX

C

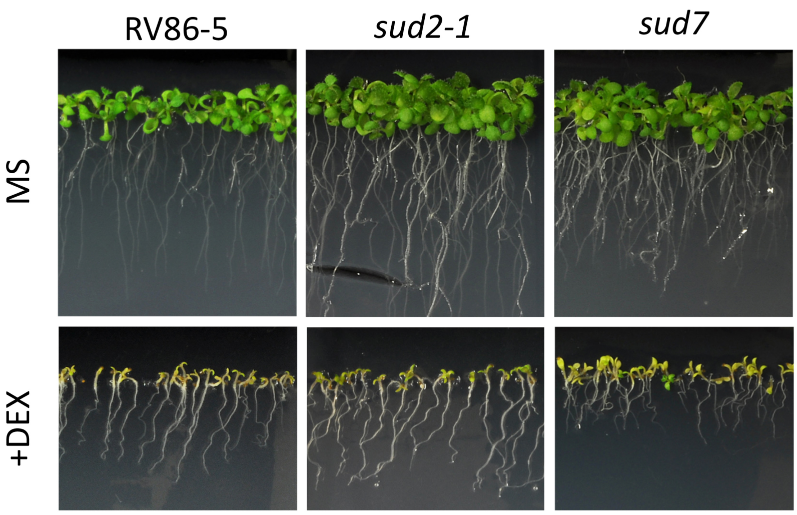

D

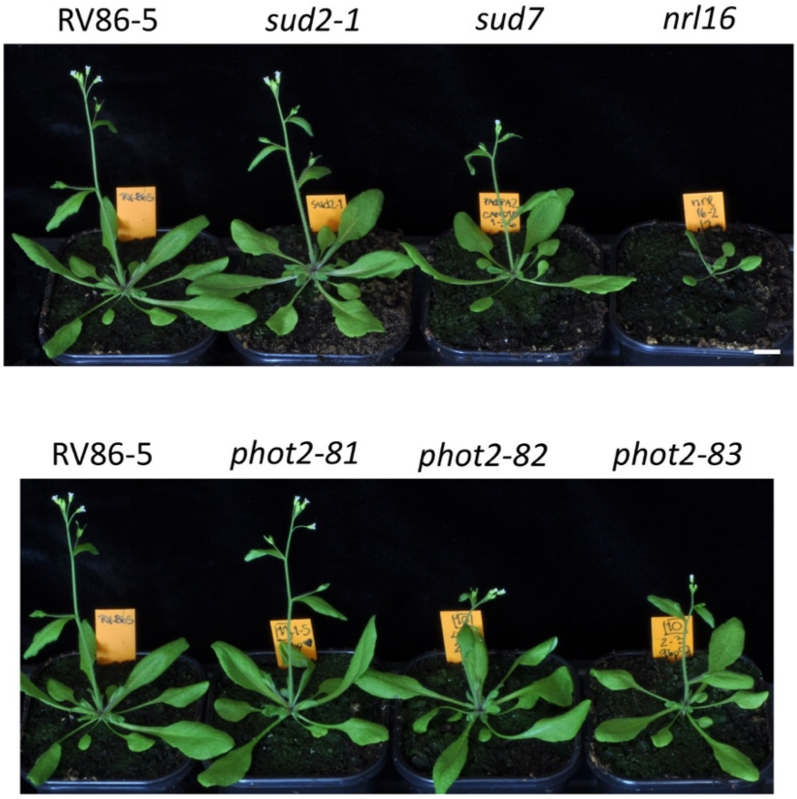

Figure S3. Growth habit of plants discussed in this work. Plants in **(A)** to **(C)** were grown on Agar plates in upright position, plants in **(D)** were soil-grown under standard long day conditions (age four weeks). Scale bar: 1 cm.

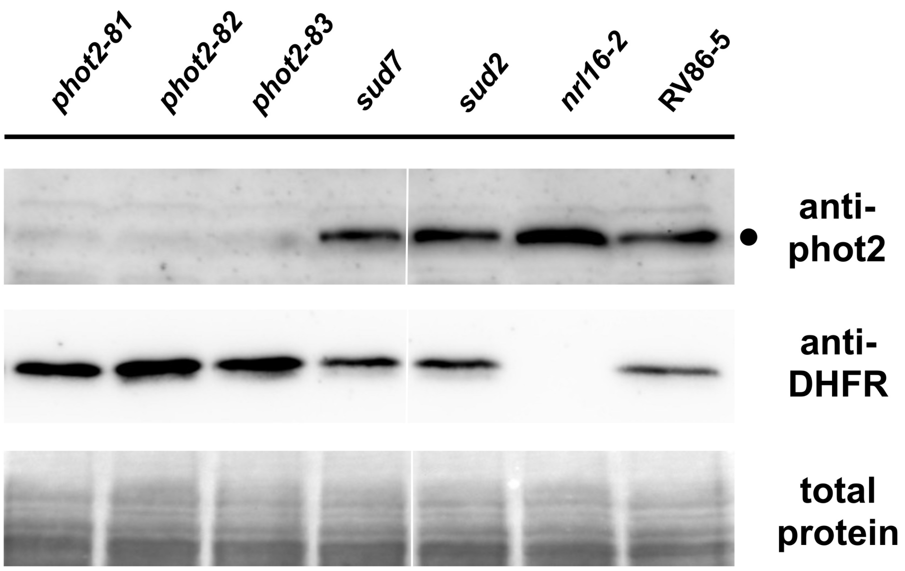

Figure S4. Western blot after induction of the ubK48R transgene by DEX in different genetic backgrounds. Levels of phot2 (dot, top panel), of DHFR as a measure of ubK48R induction (middle panel), and total protein loaded (bottom panel) were monitored. phot2 runs at ca. 110 kDa, DHFR at 23 kDa. Note that the *nrl16-2* T-DNA allele interferes with ubK48R transgene expression in absence of silencing inhibitor Zebularine. All lanes are from the same gel blot, but a lane was deleted between *sud7* and *sud2*.

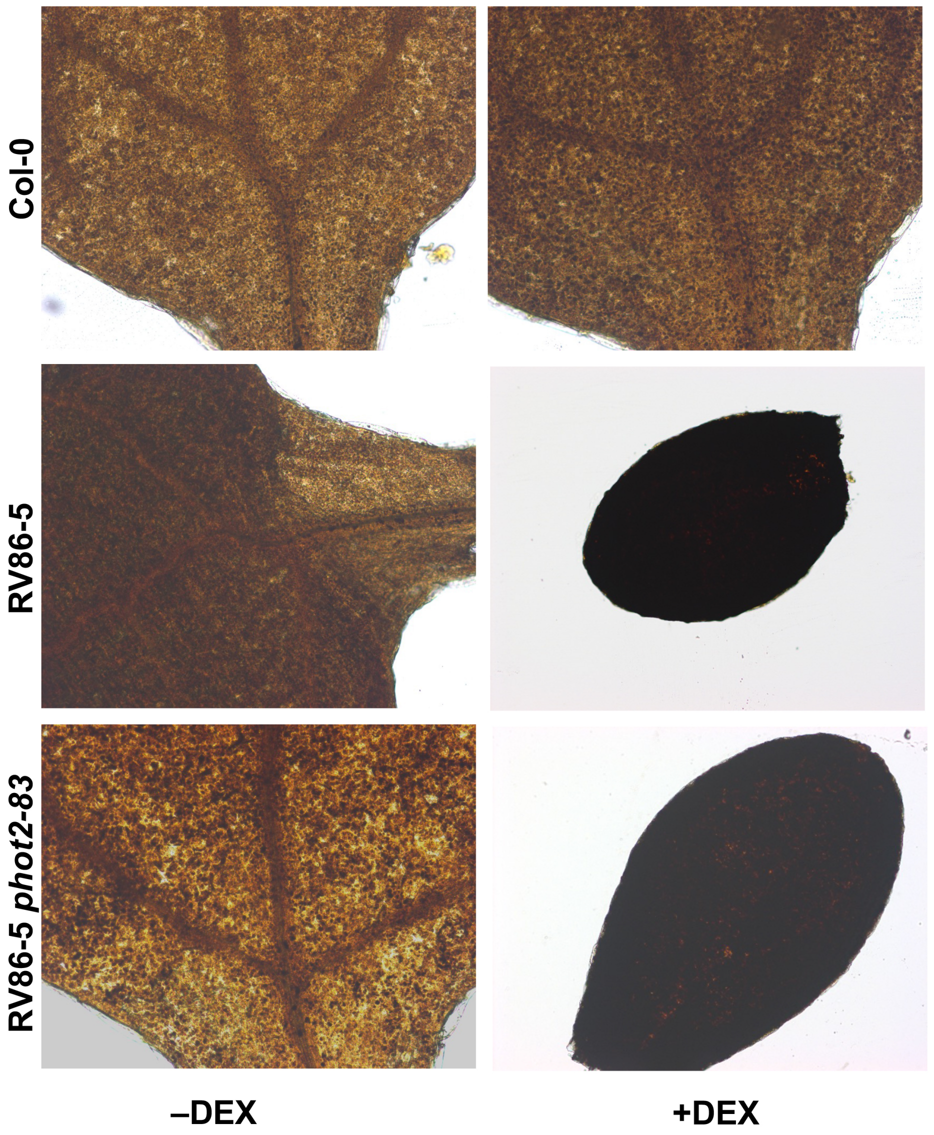

Figure S5. Lugol staining of leaves to assess starch accumulation. Leaves of seedlings exposed to Dexametasone (DEX) or un-exposed were stained to visualize starch. Due to reduced growth, leaves of RV86-5 + DEX plants are small. Leaves of the line containing the *phot2-83* suppressor allele are also enriched in starch, but do not bleach out, indicating that starch accumulation is not the cause of plant death / chloroplast destruction.

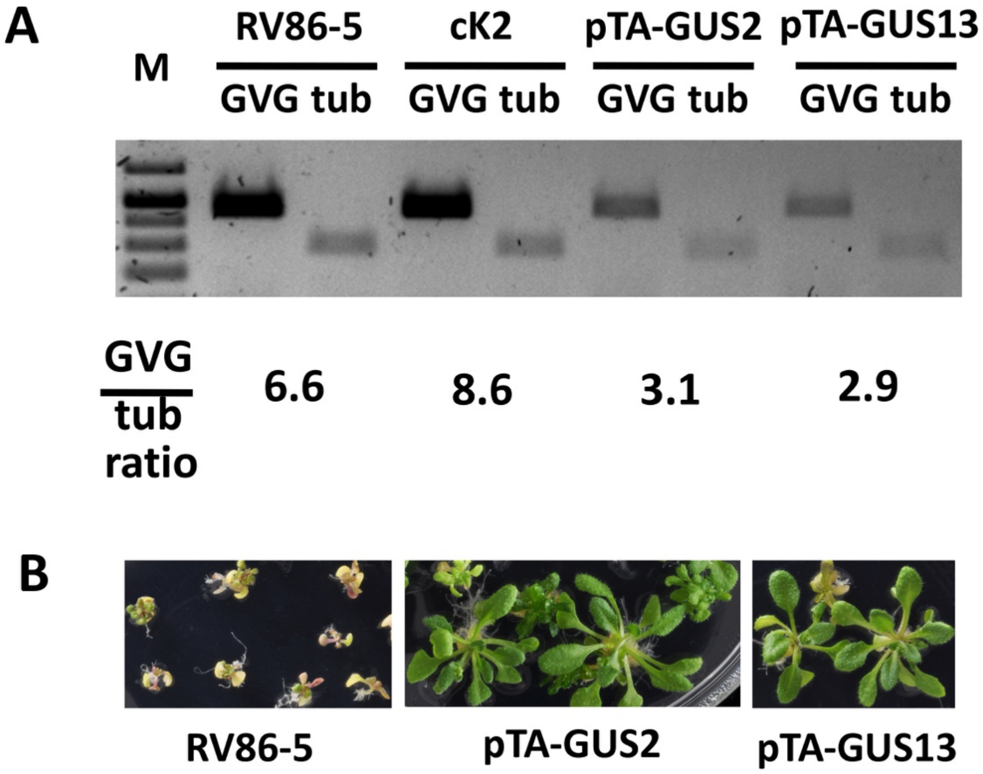

Figure S6. Growth of line RV86-5 is not impeded by its level of the GVG transcriptional activator. **(A)**, RT-PCR semi-quantitative measurement of the level of DEX-activated transcription factor GVG. Line RV86-5 was compared to line cK2 expressing a protein kinase, and to pTA-GUS lines expressing GUS instead of ubK48R with the same promoter system. Beta tubulin TUB2 (tub; At5g62690) served as a standard. Primers used for RT-PCR are listed in Table S7. Transcript levels were determined from gel images. Intensity ratios are the average of two experiments. **(B)**, Seeds of lines RV86-5, pTA-GUS2 and pTA-GUS13 were germinated in absence of inducer DEX, and transferred to DEX-containing medium after germination. Whereas RV86-5 plants uniformly arrested growth, pTA-GUS transgenic plants stayed green and continued to grow.

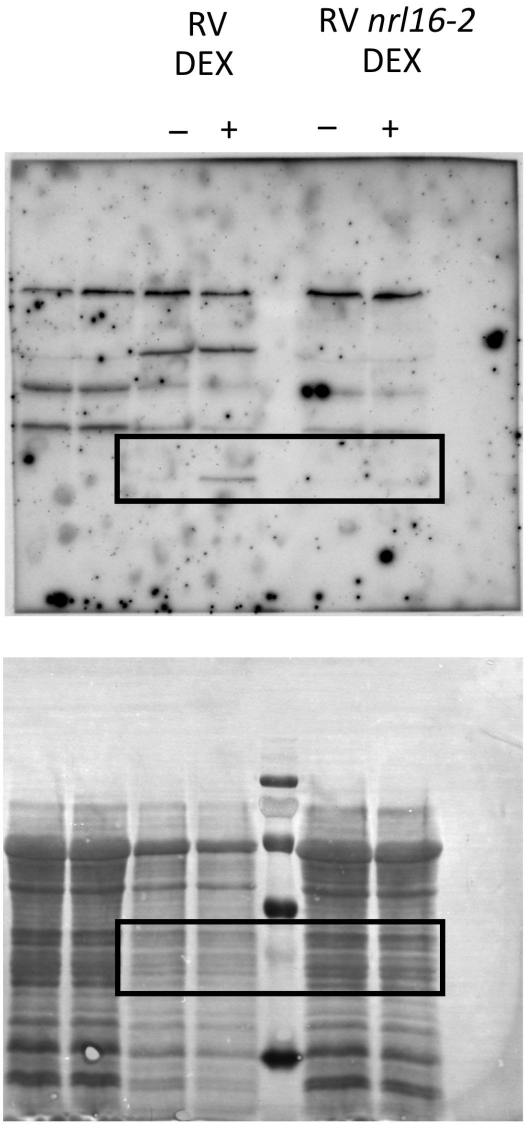

Figure S7. Whole filter of Western result shown in Figure 4. Boxed: sections included in Figure 4. Top, anti DHFR blot. Bottom, filter loading detected by the Stain-Free method (Bio Rad).

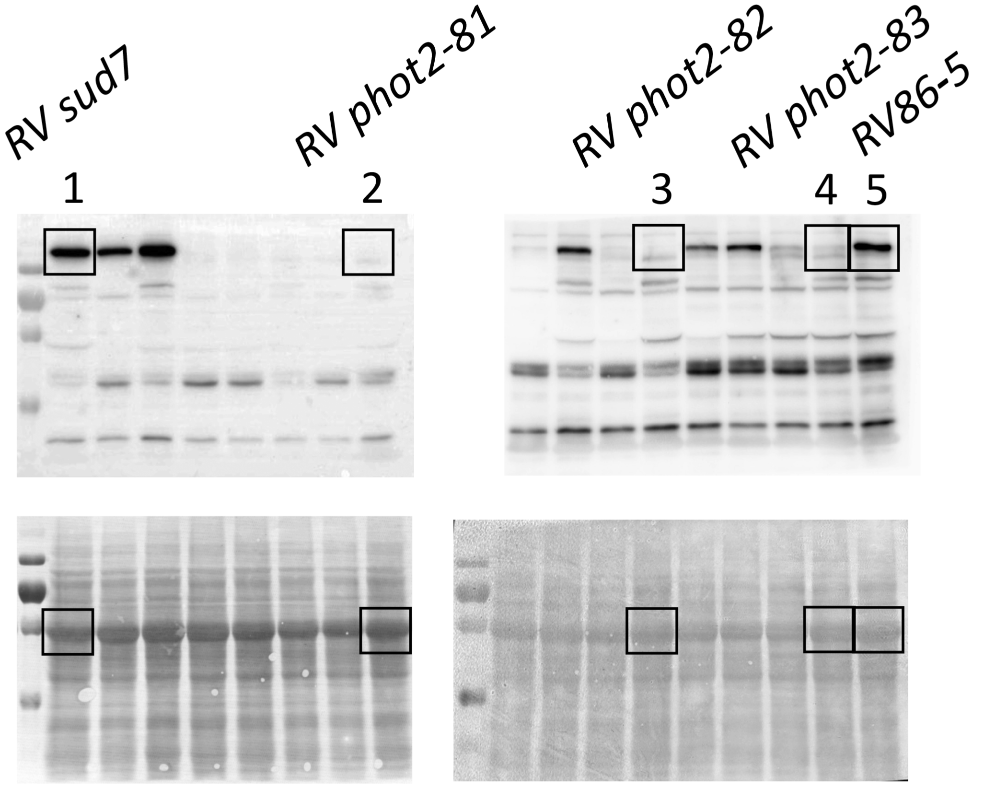

Figure S8. Whole filter of Western result shown in Figure 6C. Top, anti phototropin 2 blot. Bottom, filter loading detected by Ponceau S staining of blotted filter. Numbers indicate lane numbers of Fig. 6C, genotypes are written above. Boxed areas are shown in Figure 6C.

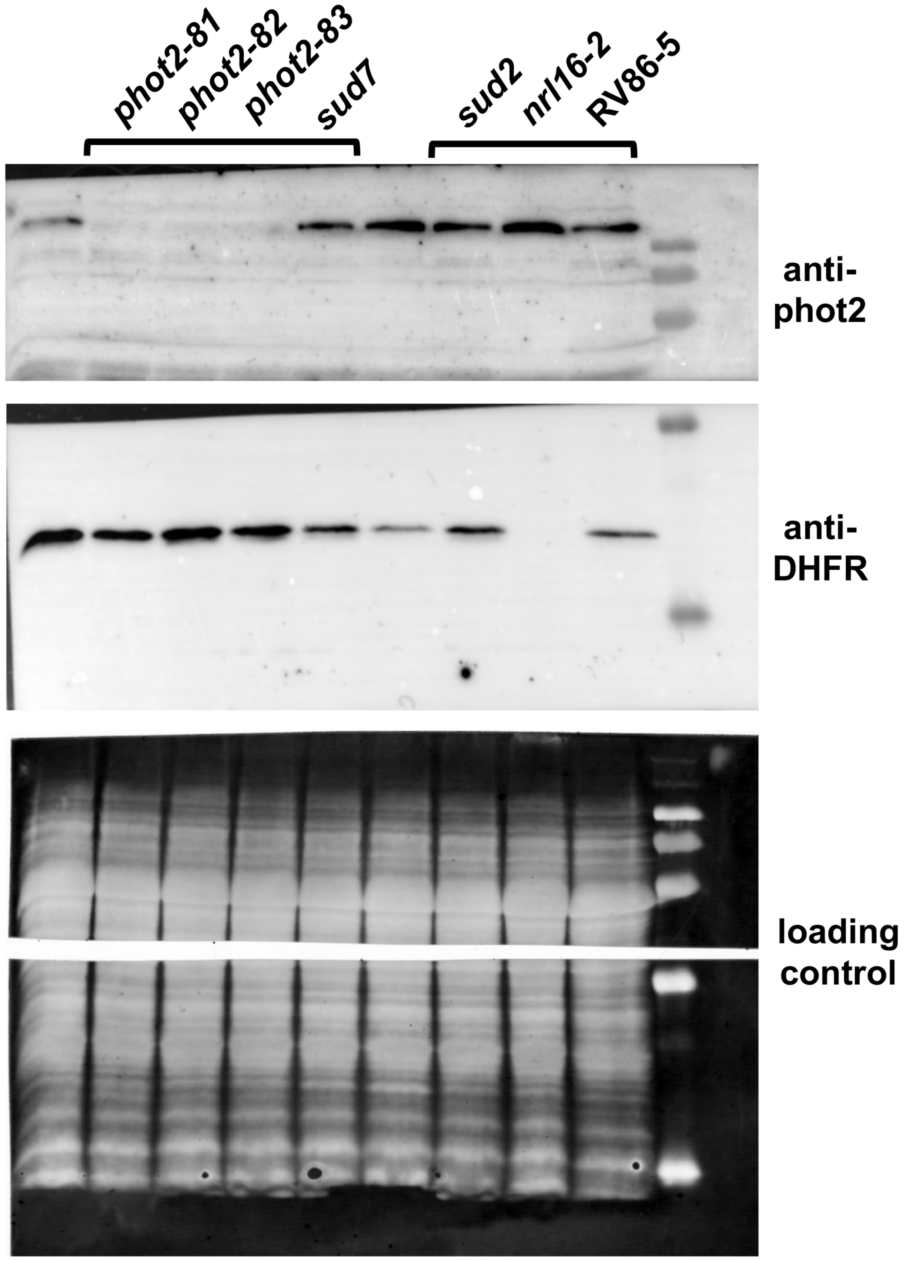

Figure S9. Whole files of Western blot shown in Figure S4. Top, anti phototropin 2 blot. Middle, anti DHFR blot. After transfer, the filter was cleaved into two parts for separate incubation with the two antibodies. Bottom, filter loading control (stain free protein detection on the filter). The brackets on top indicate parts shown in Figure S4.

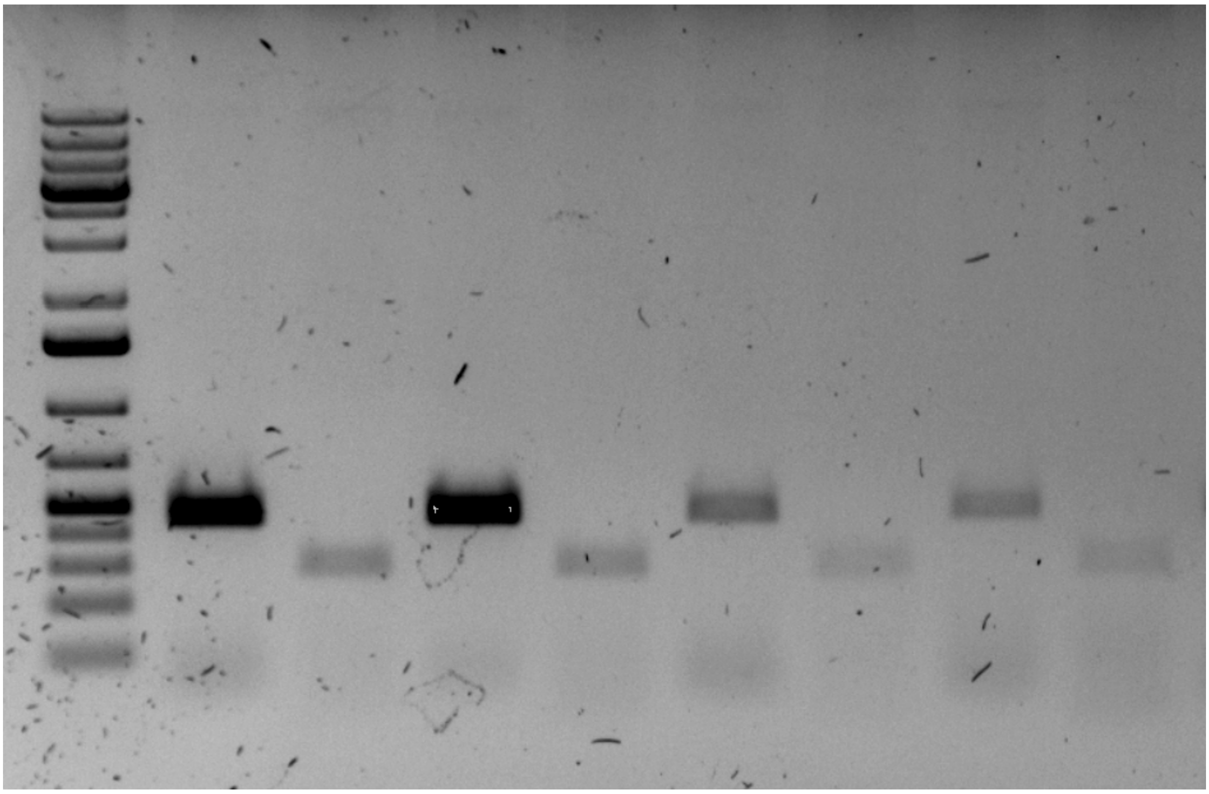

Figure S10. Whole image of agarose gel with fragments from RT-PCR reaction shown in Figure S6A.
