# Supplementary material for: Transcriptome, metabolome and suppressor analysis reveal an essential role for the ubiquitin-proteasome system in seedling chloroplast development
Source: BMC Plant Biol. 2022 Apr 8;22:183. doi: 10.1186/s12870-022-03536-6 (PMC8991883; doi:10.1186/s12870-022-03536-6)
Supplement: Supplementary file 1 — Additional file 1. [file 12870_2022_3536_MOESM1_ESM.zip › Table S3 enriched GO_2.pdf]

Genes listed in Table S1 (up-regulated):
Analysis Type: PANTHER Overrepresentation Test (Released 20200728)
Annotation Version and Release Date: GO Ontology database DOI: 10.5281/zenodo.4081749 Released 2020-10-09
Analyzed List: upload_1 (Arabidopsis thaliana)
Reference List: Arabidopsis thaliana (all genes in database)
Test Type: FISHER
Correction: BONFERRONI
Bonferroni count: 2849

GO biological process complete	Arabidopsis - thaliana REFLIST (27462)	upload_1 (632)	upload_1 (expected)	upload_1 (over/under)	upload_1 (fold Enrichment)	upload_1 (P-value)
response to wounding (GO:0009611)	226	19	5.20	+	3.65	9.46E-03
response to lipid (GO:0033993)	673	39	15.49	+	2.52	1.29E-03
interspecies interaction between organisms (GO:0044419)	1120	64	25.78	+	2.48	3.81E-07
response to oxygen-containing compound (GO:1901700)	1515	86	34.87	+	2.47	1.71E-10
response to external biotic stimulus (GO:0043207)	1093	62	25.15	+	2.46	9.42E-07
response to other organism (GO:0051707)	1093	62	25.15	+	2.46	9.42E-07
response to biotic stimulus (GO:0009607)	1094	62	25.18	+	2.46	9.64E-07
response to hormone (GO:0009725)	1241	70	28.56	+	2.45	6.61E-08
response to endogenous stimulus (GO:0009719)	1271	71	29.25	+	2.43	1.06E-07
response to external stimulus (GO:0009605)	1507	84	34.68	+	2.42	1.00E-09
transmembrane transport (GO:0055085)	855	47	19.68	+	2.39	3.53E-04
response to organic substance (GO:0010033)	1704	93	39.22	+	2.37	1.42E-10
defense response to other organism (GO:0098542)	805	42	18.53	+	2.27	7.16E-03
response to chemical (GO:0042221)	2676	137	61.58	+	2.22	6.85E-15
response to inorganic substance (GO:0010035)	913	46	21.01	+	2.19	5.54E-03
defense response (GO:0006952)	949	46	21.84	+	2.11	1.52E-02
cellular response to chemical stimulus (GO:0070887)	1084	50	24.95	+	2.00	2.24E-02
response to stimulus (GO:0050896)	5538	240	127.45	+	1.88	9.60E-21
response to abiotic stimulus (GO:0009628)	2129	90	49.00	+	1.84	1.55E-04
cell communication (GO:0007154)	1585	67	36.48	+	1.84	8.78E-03
response to stress (GO:0006950)	3100	129	71.34	+	1.81	2.09E-07
small molecule metabolic process (GO:0044281)	1516	63	34.89	+	1.81	2.98E-02
transport (GO:0006810)	2249	88	51.76	+	1.70	5.12E-03
establishment of localization (GO:0051234)	2294	88	52.79	+	1.67	1.28E-02
localization (GO:0051179)	2459	93	56.59	+	1.64	9.43E-03
cellular response to stimulus (GO:0051716)	2486	93	57.21	+	1.63	1.48E-02
regulation of biological process (GO:0050789)	5369	169	123.56	+	1.37	4.58E-02
cellular process (GO:0009987)	11931	357	274.58	+	1.30	2.60E-07
biological_process (GO:0008150)	21416	537	492.86	+	1.09	4.01E-02
Unclassified (UNCLASSIFIED)	6046	95	139.14	-	.68	0.00E00

Genes listed in Table S2 (down-regulated):
Analysis Type: PANTHER Overrepresentation Test (Released 20200728)
Annotation Version and Release Date: GO Ontology database DOI: 10.5281/zenodo.4033054 Released 2020-09-10
Analyzed List: upload_1 (Arabidopsis thaliana)
Reference List: Arabidopsis thaliana (all genes in database)
Test Type: FISHER
Correction: BONFERRONI
Bonferroni count: 2844

GO biological process complete	Arabidopsis - thaliana REFLIST (27416)	upload_1 (842)	upload_1 (expected)	upload_1 (over/under)	upload_1 (fold Enrichment)	upload_1 (P-value)
plastid transcription (GO:0042793)	11	6	.34	+	17.76	1.83E-02
rRNA modification (GO:0000154)	42	10	1.29	+	7.75	7.57E-03
maturation of LSU-rRNA (GO:0000470)	46	10	1.41	+	7.08	1.53E-02
rRNA processing (GO:0006364)	254	48	7.80	+	6.15	5.99E-18
rRNA metabolic process (GO:0016072)	263	49	8.08	+	6.07	3.87E-18
ribosomal large subunit biogenesis (GO:0042273)	109	19	3.35	+	5.68	2.47E-05
response to karrikin (GO:0080167)	128	20	3.93	+	5.09	5.22E-05
ribosome biogenesis (GO:0042254)	370	56	11.36	+	4.93	3.08E-17
ncRNA processing (GO:0034470)	382	54	11.73	+	4.60	2.36E-15
chloroplast organization (GO:0009658)	235	30	7.22	+	4.16	1.29E-06
ribonucleoprotein complex biogenesis (GO:0022613)	447	57	13.73	+	4.15	1.78E-14
ncRNA metabolic process (GO:0034660)	456	57	14.00	+	4.07	4.02E-14
protein folding (GO:0006457)	181	22	5.56	+	3.96	5.78E-04
plastid organization (GO:0009657)	306	36	9.40	+	3.83	1.90E-07
photosynthesis (GO:0015979)	192	22	5.90	+	3.73	1.44E-03
response to heat (GO:0009408)	226	24	6.94	+	3.46	1.57E-03
response to wounding (GO:0009611)	216	21	6.63	+	3.17	2.76E-02
response to temperature stimulus (GO:0009266)	615	56	18.89	+	2.96	1.47E-08
response to cold (GO:0009409)	413	36	12.68	+	2.84	2.66E-04
RNA processing (GO:0006396)	791	63	24.29	+	2.59	1.43E-07
response to oxidative stress (GO:0006979)	455	34	13.97	+	2.43	1.57E-02
response to light stimulus (GO:0009416)	712	53	21.87	+	2.42	4.50E-05
response to radiation (GO:0009314)	737	54	22.63	+	2.39	7.59E-05
cellular component biogenesis (GO:0044085)	1321	94	40.57	+	2.32	1.25E-09
RNA metabolic process (GO:0016070)	1299	92	39.89	+	2.31	2.78E-09
response to abiotic stimulus (GO:0009628)	2126	150	65.29	+	2.30	3.83E-17
gene expression (GO:0010467)	1608	112	49.38	+	2.27	1.78E-11
cellular nitrogen compound biosynthetic process (GO:0044271)	1233	76	37.87	+	2.01	1.11E-04
response to hormone (GO:0009725)	1237	74	37.99	+	1.95	4.82E-04
response to endogenous stimulus (GO:0009719)	1267	74	38.91	+	1.90	1.07E-03
response to oxygen-containing compound (GO:1901700)	1504	86	46.19	+	1.86	3.27E-04
nucleic acid metabolic process (GO:0090304)	1709	97	52.49	+	1.85	4.86E-05
response to organic substance (GO:0010033)	1699	94	52.18	+	1.80	3.40E-04
response to stress (GO:0006950)	3089	169	94.87	+	1.78	1.13E-09

nucleobase-containing compound metabolic process (GO:0006139)	2089	111	64.16	+	1.73	1.29E-04
cellular nitrogen compound metabolic process (GO:0034641)	2869	151	88.11	+	1.71	4.43E-07
response to chemical (GO:0042221)	2674	140	82.12	+	1.70	3.61E-06
response to external stimulus (GO:0009605)	1509	79	46.34	+	1.70	2.53E-02
heterocycle metabolic process (GO:0046483)	2389	124	73.37	+	1.69	6.90E-05
organic cyclic compound metabolic process (GO:1901360)	2604	135	79.97	+	1.69	1.45E-05
cellular aromatic compound metabolic process (GO:0006725)	2516	129	77.27	+	1.67	7.23E-05
cellular component organization or biogenesis (GO:0071840)	2807	143	86.21	+	1.66	1.18E-05
response to stimulus (GO:0050896)	5562	283	170.82	+	1.66	2.27E-15
organic substance biosynthetic process (GO:1901576)	2659	122	81.66	+	1.49	3.66E-02
biosynthetic process (GO:0009058)	2797	128	85.90	+	1.49	2.54E-02
cellular metabolic process (GO:0044237)	7620	299	234.03	+	1.28	4.48E-03
cellular process (GO:0009987)	11983	462	368.02	+	1.26	5.49E-07
metabolic process (GO:0008152)	8683	331	266.67	+	1.24	1.27E-02
Unclassified (UNCLASSIFIED)	5454	133	167.50	-	.79	0.00E00
