# Supplementary material for: Transcriptome, metabolome and suppressor analysis reveal an essential role for the ubiquitin-proteasome system in seedling chloroplast development
Source: BMC Plant Biol. 2022 Apr 8;22:183. doi: 10.1186/s12870-022-03536-6 (PMC8991883; doi:10.1186/s12870-022-03536-6)
Supplement: Supplementary file 1 — Additional file 1. [file 12870_2022_3536_MOESM1_ESM.zip › Table S7_2 Oligonucleotides used in this work.pdf]

Table S7 Oligonucleotides used in this work

2431-NPH3pGBKdn1	Oligo for cloning of the NPH3 ORF into Vector pGBKT7	GGA GGC CGA ATT CCC TAT GAT GTG GGA ATC TGA GA
2432-NPH3pGBKup1	as 2431	GGT CGA CGG ATC CCC TCA TGA AAT TGA GTT CCT CCA
2433-SUD8pGBKdn1	Oligo for cloning of the NRL16 ORF into Vector pGBKT7	GGA GGC CGA ATT CCC TAT GTC ACC TGT TGC TAA AGT
2434-SUD8pGBKup1	as 2433	GGT CGA CGG ATC CCC TTA TCC GAA GAA AGA GAA GCT
2435-SUD8pGADTdn1	Oligo for cloning of the NRL16 ORF into Vector pGADT7	TGG CCA TTA TGG CCC ATG TCA CCT GTT GCT AAA GT
2436-SUD8pGADTup1	as 2435	GAC ATG TTT TTT CCC TTA TCC GAA GAA AGA GAA GCT
2445-NPH3pGADTdn1	Oligo for cloning of the NPH3 ORF into Vector pGADT7	TGG CCA TTA TGG CCC ATG ATG TGG GAA TCT GAG A
2446-NPH3pGADTup1	as 2445	GAC ATG TTT TTT CCC TCA TGA AAT TGA GTT CCT CCA
2447-PHOT2pGADTdn1	Oligo for cloning of the Phototropin 2 ORF into Vector pGADT7	TGG CCA TTA TGG CCC ATG GAG AGG CCA AGA GCC
2448-PHOT2pGADTup1	as 2447	GAC ATG TTT TTT CCC TTA GAA GAG GTC AAT GTC C
2449-PHOT2pGBKdn1	Oligo for cloning of the Phototropin 2 ORF into Vector pGBKT7	GGA GGC CGA ATT CCC ATG GAG AGG CCA AGA GCC

2450-PHOT2pGBKup1	as 2449	GGT CGA CGG ATC CCC TTA GAA GAG GTC AAT GTC C
1751-CRISphoSDM3p	Oligo for insertion of sgRNA into vector pCHIMERA via site-directed mutagenesis	GAA GTA GTG ATT GAC TCC TAT CAA GGA CGA CCA GTT TTA GAG CTA GA
1752-CRISphoSDM3m	Complementary oligo to 1751	CTA GCT CTA AAA CTG GTC GTC CTT GAT AGG AGT CAA TCA CTA CTT CG
1643-PHOT2dn5	Oligo for amplification of mutated region of PHOT2	TCC ATC TCC TTT GAA TGA TGC
1644-PHOT2up8	Second oligo for amplification of mutated region of PHOT2	AGT GTC ATT GCT CAC GGA TTC
1485-GVG1	Oligo for RT-PCR to quantify GVG transcriptional activator mRNA	GGG GGA GCT CAT GAA GCT ACT GTC TTC TAT
1486-GVG2	Second oligo for GVG transcriptional activator mRNA detection via RT-PCR	TTT ATT AAC TCT TAT CCA TCC ATT TGC
664A-tub1	RT-PCR control tubulin mRNA quantification	ACT CGT TGG GAG GAG GAA CT
665A-tub2	Second oligo for RT-PCR control mRNA quantification (tubulin)	ACA CCA GAC ATA GTA GCA GAA ATC AAG
